# Introns Regulate Gene Expression in *Cryptococcus neoformans* in a Pab2p Dependent Pathway

**DOI:** 10.1371/journal.pgen.1003686

**Published:** 2013-08-15

**Authors:** Carolin Goebels, Aline Thonn, Sara Gonzalez-Hilarion, Olga Rolland, Frederique Moyrand, Traude H. Beilharz, Guilhem Janbon

**Affiliations:** 1 Institut Pasteur, Unité des Aspergillus, Département Parasitologie et Mycologie, Paris, France; 2 Monash University, Department of Biochemistry and Molecular Biology, Clayton, Australia; University of California San Francisco, United States of America

## Abstract

Most *Cryptococccus neoformans* genes are interrupted by introns, and alternative splicing occurs very often. In this study, we examined the influence of introns on *C. neoformans* gene expression. For most tested genes, elimination of introns greatly reduces mRNA accumulation. Strikingly, the number and the position of introns modulate the gene expression level in a cumulative manner. A screen for mutant strains able to express functionally an intronless allele revealed that the nuclear poly(A) binding protein Pab2 modulates intron-dependent regulation of gene expression in *C. neoformans*. *PAB2* deletion partially restored accumulation of intronless mRNA. In addition, our results demonstrated that the essential nucleases Rrp44p and Xrn2p are implicated in the degradation of mRNA transcribed from an intronless allele in *C. neoformans*. Double mutant constructions and over-expression experiments suggested that Pab2p and Xrn2p could act in the same pathway whereas Rrp44p appears to act independently. Finally, deletion of the *RRP6* or the *CID14* gene, encoding the nuclear exosome nuclease and the TRAMP complex associated poly(A) polymerase, respectively, has no effect on intronless allele expression.

## Author Summary

*Cryptococcus neoformans* is a major human pathogen responsible for deadly infection in immunocompromised patients. The analysis of its genome previously revealed that most of its genes are interrupted by introns. Here, we demonstrate that introns modulate gene expression in a cumulative manner. We also demonstrate that introns can play a positive or a negative role in this process. We identify a nuclear poly(A) binding protein (Pab2p) as implicated in the intron-dependent control of gene expression in *C. neoformans*. We also demonstrate that the essential nucleases Rrp44p and Xrn2p are implicated in two independent pathways controlling the intron-dependent regulation of gene expression in *C. neoformans*. Xrn2p regulation seems to depend on Pab2p whereas Rrp44p acts independently. In contrast, the other exosome nuclease Rrp6p and the TRAMP associated poly(A) polymerase Cid14p do not appear to be implicated in this regulation. Our results provide new insights into the regulation of gene expression in eukaryotes and more specifically into the biology and virulence of *C. neoformans*.

## Introduction

Introns, discovered in 1977, are genomic sequences that are removed from the corresponding RNA transcripts of genes [1]. First considered just as elements to be removed for correct gene expression, it has since become obvious that they participate in many aspects of gene regulation. Actually, the presence of introns and their splicing by the RNA-protein complex named spliceosome [2] affect gene expression by different means [3] including transcription, polyadenylation, mRNA export, mRNA localisation, translation efficiency and the rate of mRNA decay (see [4] for review). Most eukaryotic genes contain introns although the proportion of genes containing introns is highly variable between organisms. For example, whereas 92% and 78% of the genes in human and plant genomes contain introns, respectively, [5], [6] introns are found in only 5% of the genes in the yeast *Saccharomyces cerevisiae* [7]. Furthermore, the influence of introns on gene expression differs from one organism to another and from one gene to another.

In mammals, the expression of most of the genes is reduced in the absence of splicing but the effect of introns on gene expression is generally modest [8]. In contrast, the expression of some genes like the β-*globin* gene or the purine nucleoside phosphorylase gene has been shown to be highly intron-dependent [9], [10]. Introns act mainly at a post-transcriptional level and their absence reduces nuclear and cytoplasmic mRNA accumulation, alters efficient mRNA 3′end formation and consequently reduces nuclear mRNA export [8], [11], [12]. Introns seem also to regulate mRNA translation efficiency [8], [11], [12]. Similarly in plants most mutations can be complemented by cDNA sequences suggesting that most genes do not require introns for expression. For a few genes however, IME (intron-mediated enhancement) of gene expression has been demonstrated [13]. IME has been shown to act at a post transcriptional level and to be, at least for some genes, independent of splicing *per se* [14], [15]. More recently, IME has been shown to regulate 3′UTR formation and, to a lesser extent, translation [15]. However, in both plants and mammals, the pathway by which mRNAs transcribed from intronless alleles are degraded has not been described [16].

In fungi, the information is even more sparse, with most data coming from studies on *S. cerevisiae*, in which introns are rare and generally not necessary for gene expression [17], [18]. In a few examples however, introns have been shown to be necessary for gene expression, controlling the export of mRNA from the nucleus [19], [20]. More recently, introns have been shown to be key modulators of ribosomal protein gene expression in the baker's yeast [21]. In the other hemiascomycete yeasts in which the percentage of intron containing genes goes from 2.4% in *Candida glabrata* to 14.5% in *Yarrowia lipolytica* [22], introns do not seem to be necessary for gene expression although no specific studies have been reported. Similarly, in *Schizosaccharomyces pombe* in which 47% of the genes contain introns [23], these are generally not necessary for gene expression [24]. In filamentous fungi like *Aspergillus nidulans* or *Neurospora crassa*, cDNA sequences have been widely used for the production of heterologous or homologous proteins [25]–[27] suggesting only a moderate influence of introns on gene expression. In two cases however, one in *Podospora anserina* and one in *Trichoderma viride*, introns were reported to be necessary for gene expression but the mechanisms by which this regulation occurs have not been studied [28], [29]. Similarly in basidiomycetes, although intron density is generally higher than in ascomycetes [30], only a few cases of alteration of gene expression by the elimination or the addition of introns have been described [31]–[35]. In *Schizophyllum commune*, the addition of one intron in a *GFP* reporter gene has been shown to increase gene expression by altering mRNA accumulation rather than the level of transcription although no further description of the mechanisms by which this regulation occurs has been reported [31].

*Cryptococcus neoformans* is a capsular basidiomycete yeast mainly studied because it is responsible for opportunistic infections in patients presenting a cellular immune deficiency (mainly AIDS patients) that are fatal if left untreated [36]. The presence of an antiphagocytic polysaccharide capsule and the production of the antioxidant melanin are its two major virulence factors [37], [38]. The genome (20 Mb) sequences of five strains, two of serotype D, one of serotype A, and two of serotype B are now complete [39], [40]. The sequences of the 14 chromosomes of the serotype D strains were annotated using 21000 cDNA sequences isolated from a normalized library. Of the 6574 predicted genes, 80% had confirmed transcripts associated with them. Interestingly, *C. neoformans* genes are intron-rich and more than 98% of them have been reported to contain introns. Thus, *C. neoformans* has probably the intron-richest annotated genome described to date. These introns (5 on average per gene) are very small in size (67 bp) whereas exons have a size (250 bp) close to the human ones [40], [41].). Alternative splicing has been reported to be very common in *C. neoformans* and intron retention represents its most common manifestation [40], [42]. Finally, the fact that the proteome of *C. neoformans* contains numbers of proteins sharing sequence similarities with known metazoan SR proteins ([43]; Janbon unpublished data) as well as the identification of a DEAD-box helicase as a central regulator of multiple virulence factors [44] suggest that intron-dependent regulation of gene expression might play a major role in *C. neoformans* biology and virulence.

In this article, we have addressed the importance of introns for gene expression in *C. neoformans*. We have shown that introns are necessary for mRNA accumulation for some genes but not for others. We also demonstrated that introns can play a positive or a negative role in this process. Finally, we showed that the nuclear poly(A) binding protein Pab2 and the exosome nuclease Rrp44p are implicated in this intron-dependent regulation of gene expression in *C. neoformans*. Our results also suggested that Xrn2p might act in the same pathway as Pab2p.

## Results

### Evidence for intron retention at the *CAS3* locus

**Figure 1:**
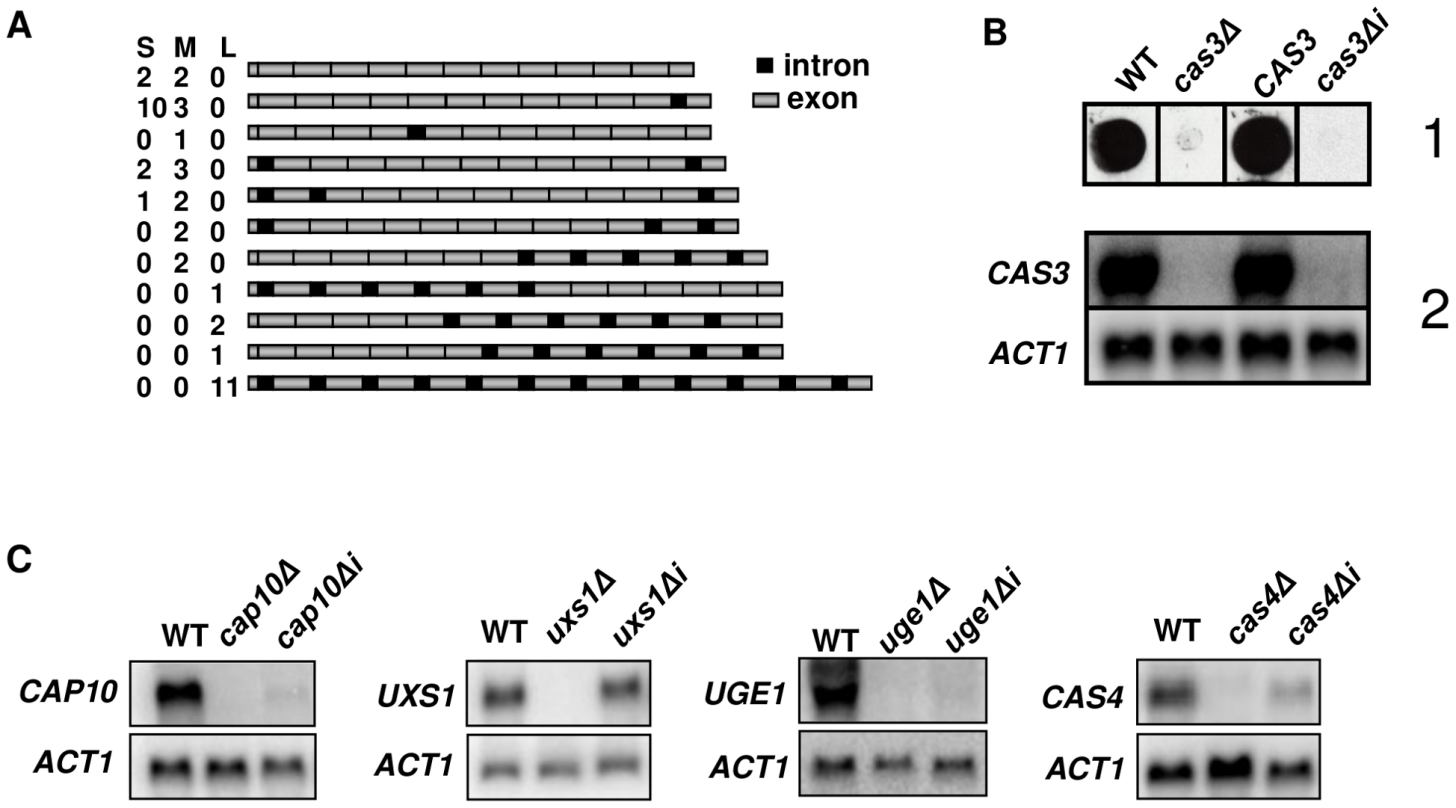
Influence of introns on gene expression. **A**. Schematic representation of the different cDNA molecules sequenced illustrating intron retention in *CAS3* mRNA. *CAS3* specific cDNA molecules were RT-PCR-amplified from purified mRNA, separated in three pools according to their sizes (Small, Medium and Large) and cloned. Fifteen clones were sequenced per pool and the presence of introns was analyzed. The numbers of clones obtained for each isoform in each pool are indicated. Introns and exons are not at real scale. **B**. *cas3Δi* is a non-functional allele. When the *cas3Δ* allele was replaced by the intronless allele, there were very little *CAS3* mRNAs and no complementation of the capsule phenotype. (1) Antibody reactivity of the GXM-specific Mab CRND-8 with the mutant strains. 10^4^ cells were spotted on a nitrocellulose membrane and probed with CRND-8. (2) Northern blot experiment results showing very low mRNA level of the *cas3Δi* allele. **C**. Intron-dependent regulation of mRNA accumulation is gene specific. Two independently obtained *Δi* strains were tested here giving identical results (data not shown). As a control, we checked that replacing the *cas3Δ* allele by the wild type gene (*CAS3*) using the same procedure restored the antibody reactivity and the level of mRNA accumulation.

We previously reported that the *CAS3* gene contains 12 introns, all of them but the last one (intron 12) being located within the CDS [45]. We performed RACE experiments and noticed that among the five 5′end cDNAs sequenced, two were copies of RNA molecules not spliced in the intron 1 whereas the intron 2 was spliced. In order to identify the different types of *CAS3* mRNA molecules present in the cell we sequenced a large number of full length cDNAs. Poly(A) RNA molecules were purified from *C. neoformans* var. *neoformans* cells growing in YPD and used for RT-PCR experiments. After separation by gel electrophoresis and purification, 3 pools of 15 full length cDNA molecules were cloned and sequenced (see Material and Methods). As presented in Figure 1A, a large diversity of *CAS3* RNA molecules was identified, ranging from completely spliced molecules to completely unspliced ones. Although these experiments were not quantitative, the pattern of splicing observed revealed that some introns were more rarely spliced than others. Introns 1 and 12 were spliced in only 51% and 18% of the 45 sequenced molecules, respectively. RNA-Seq data alignment pattern analysis confirmed that all the introns from this gene can display a certain level of intron retention (Janbon, unpublished data). With the obvious exception of intron 12 which lies in the 3′UTR, all introns of this gene contain at least one in frame stop codon suggesting that none of these intron-containing mRNA molecules could encode a protein.

### Introns are necessary for *CAS3* expression

Evidence for intron retention at the *CAS3* locus suggested that at least part of the regulation of this transcript was dependent on introns. So as to analyse the influence of intronic sequences on *CAS3* expression we replaced the *CAS3* wild type allele by a version without introns (*cas3Δi*). The co-transformation procedure used here allowed a complete allele replacement at its original locus without any further modification of the local genomic landscape (see Material and Methods). The main phenotype associated with the deletion of *CAS3* is a modification of the capsule structure that can be revealed using anti-capsule monoclonal antibodies [46]. As shown in Figure 1B, the capsule structure of the strain bearing *cas3Δi* was similar to the one in which the gene had been deleted (*cas3Δ*) suggesting that the intronless allele was not functional. Moreover, Northern blot experiments showed that very little *CAS3* specific RNA was present in the *cas3Δi* strain (Figure 1B). Introns are thus necessary for *CAS3* expression.

To verify whether the importance of introns on gene expression was a general feature in *C. neoformans*, we cloned cDNAs from the genes *UXS1*, *CAP10*, *UGE1* and *CAS4* under the control of their own promoter. These constructs were used to transform the corresponding deletion mutant strains. We then compared mRNA levels of intronless and wild type alleles by Northern blot analysis. As presented in Figure 1C, the influence of introns on mRNA level was gene-dependent. Some genes, like *CAP10*, *UGE1* and to a lesser extend *CAS4* were highly intron-dependent whereas others like *UXS1* did not depend on the presence of such sequences to be expressed.

### *CAS3* introns act at a post-transcriptional level

The intron-dependent regulation of mRNA accumulation could act at different levels. Thus, the absence of RNA in the *cas3Δi* strains could be due to the absence or a very low level of transcription or/and to a decrease of the stability of the corresponding RNA leading to a complete or nearly complete degradation of it. To answer this question, we performed nuclear run-on experiments, thus measuring the frequency of transcription initiation of the different alleles largely independently of the effects of RNA stability [47]. The ratio of the *CAS3* specific signal versus the *ACT1* specific was not altered by the absence of introns (1.26±0.25) when compared to the wild-type allele (1.29±0.47) (Figure S1). These results suggested that the absence of introns does not alter the transcriptional activity of the gene but rather greatly alters the stability of RNA molecules transcribed from the *cas3Δi* allele.

### Introns influence mRNA accumulation in *C. neoformans* positively and negatively

**Figure 2:**
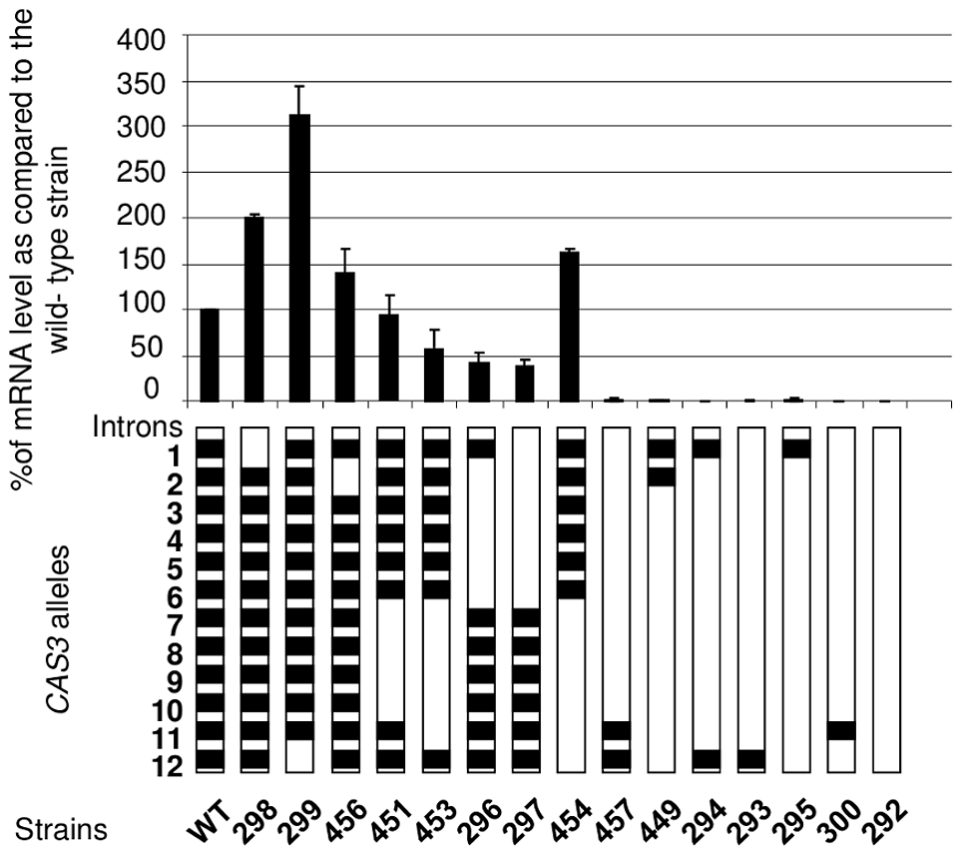
Intron number and position influence mRNA level. Different *CAS3* alleles bearing different numbers and positions of introns were introduced at the original *CAS3* locus and the level of *CAS3* mRNA was measured by RT qPCR. The reported values are the means ± SD of three independent experiments.

So as to better analyse the influence of the introns on gene expression, we constructed a series of alleles bearing different numbers of introns at different positions. These alleles were integrated at the wild-type locus following the same procedure used previously. The level of mRNA was then measured by Northern analysis and confirmed by RT-qPCR (Figure 2). As previously shown, in the absence of introns, very few transcripts can be detected (less than 1% of the wild-type). Surprisingly, the presence of one intron was not enough to restore any expression of the gene as demonstrated by the analysis done with the strains NE293, NE295 and NE300 where the introns 12, 1, or 11 were present, respectively. Even with 2 introns, the expression of the gene remained barely detectable (see strains NE294, NE457 and NE449). The intron 12 and to a lesser extent the intron 1 appeared to play a negative regulatory role in *CAS3* expression. Indeed, in strains NE298 and NE299 in which the *CAS3* allele lacks the intron 1 and 12, respectively, the expression of this gene went up 2–3 fold. The negative role of the intron 12 in *CAS3* gene expression was confirmed by comparing the expression of the *CAS3* alleles from the strains NE454 (without intron 12) and NE453 (with intron 12). By comparison the absence of intron 2 influenced poorly the level of expression of the gene (strain NE456). The presence of the other introns regulated *CAS3* expression in a positive way. In fact, excepting the regulation by introns 1 and 12, the more introns were present in *CAS3* the better the gene was expressed. The identity of the introns did not seem to be important. Indeed, deletion of introns 2 to 6 (strain NE296) altered *CAS3* mRNA level to the same degree as the deletion of introns 7 to 11 (NE453). Accordingly, the negative effect of the intron 12 appeared to be more dependent on its position than on its sequence as the re-positioning of the intron 2 at the intron 12 position in the *cas3Δi12* allele results in a wild type expression of this intron swapped allele (127% of mRNA accumulation as compared to the wild type) (Figure S2).

Finally, dot blot assays using an anti-capsule antibody were performed to see whether *CAS3* mRNA levels correlated with the phenotype of the corresponding strains. Results shown in Figure S3 demonstrated that a level of expression of *CAS3* of at least 37% (Figure 2) of the wild-type mRNA level is associated with a wild-type capsule phenotype.

### Pab2p regulates intronless allele expression

We aimed to identify elements involved in the degradation of the RNA molecule transcribed from the intronless allele *cas3Δi*. We constructed and screened an insertional library of *C. neoformans* mutants using a dot blot assay and the anti-capsule monoclonal antibody CRND-8. More than 5000 mutant strains were tested and fourteen strains were identified as having a low but detectable reactivity with this antibody (data not shown). Northern blot analysis confirmed that all of them expressed the *cas3Δi* allele at a low level (data not shown). Analysis of the position of the insertion site in the first mutant strain studied, revealed that it was within the gene CNB04570 coding for a protein of 210 amino acids sharing 49% and 32% of amino acid sequence identity with the nuclear poly (A) binding protein of *S. pombe* [48] and the human one, respectively [49]. Like its human and fission yeast counterparts, the *C. neoformans* Pab2p sequence presented a single RNA binding domain and an arginine-rich C-terminal domain. However, the poly-alanine domain present in the human protein in which mutations associated with the genetic disease named Oculopharyngeal muscular dystrophy (OPMD) have been identified [49], was absent in both fungal proteins.

**Figure 3:**
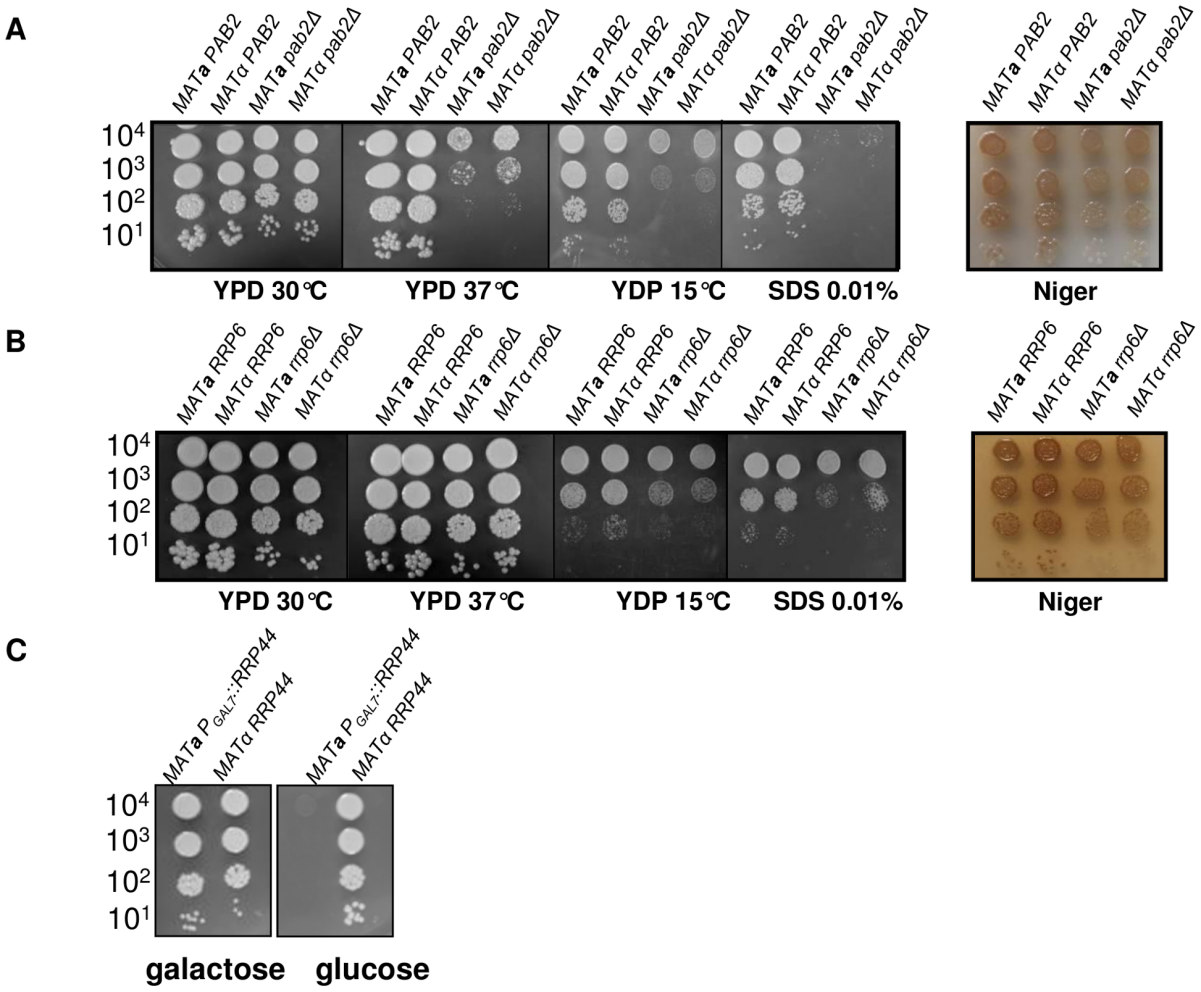
Growth phenotypes associated with *PAB2* (panel A), *RRP6* (panel B) and *RRP44* (panel C) mutations. Serial dilutions of cells were spotted on different media. Pictures were taken after three days of incubation.

We deleted *PAB2* using a nourseothricin marker (see Material and Methods). As previously reported in *S. pombe*, the *pab2Δ* mutants grow less well at 15°C as compared to the wild-type strains. In *C. neoformans*, we also found that this mutation results in an alteration of the growth rate at 30°C and 37°C (Figure 3A) and an increased sensitivity to SDS 0.01% as compared to the wild-type. We also studied the classically associated virulence phenotypes and found no evidence of modification of the capsule size or structure and no alteration of the urease production (data not shown). In contrast, we observed a small but reproducible reduction in melanin production (Figure 3A).

**Figure 4:**
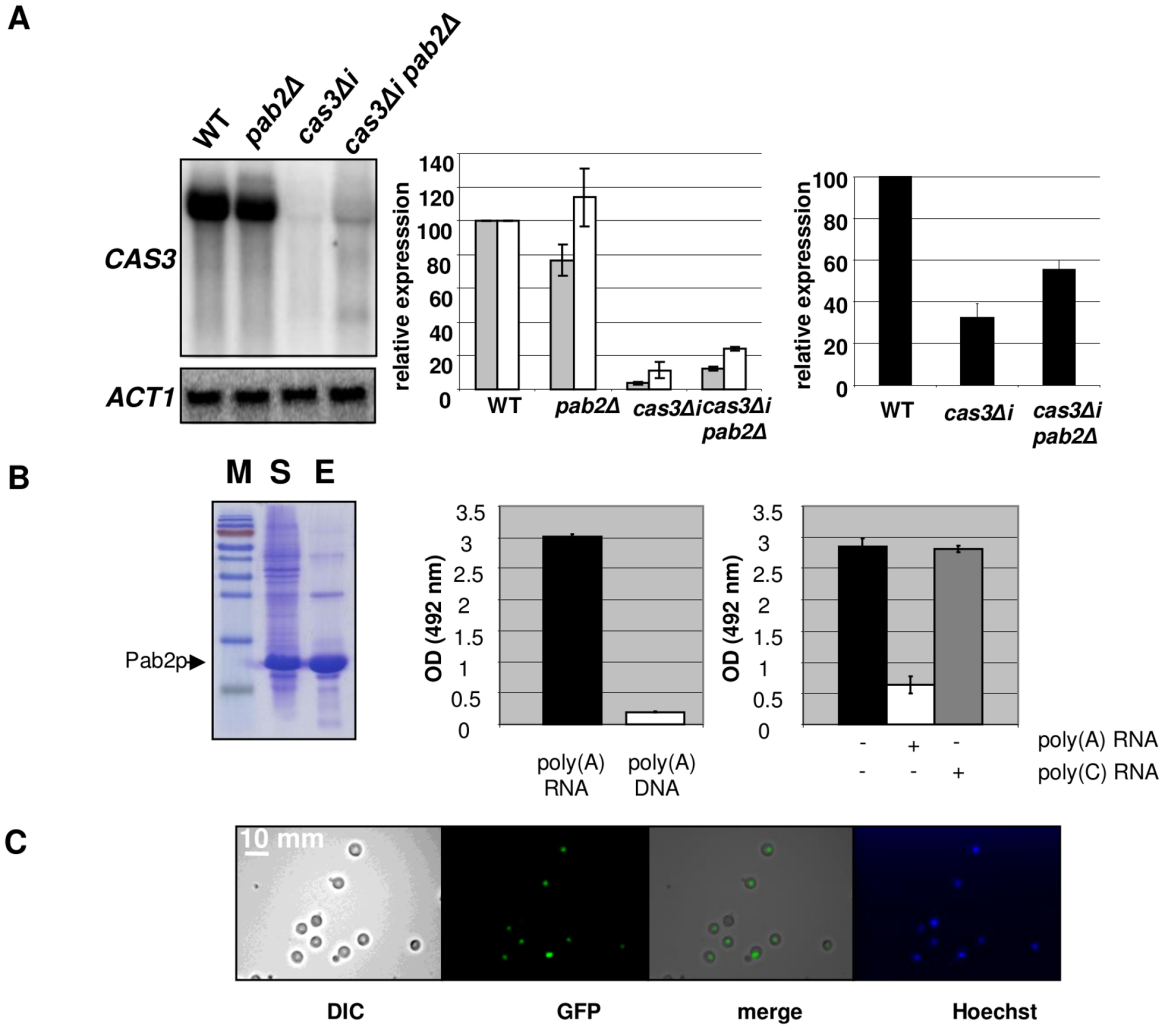
Pab2p is a nuclear poly(A) binding protein necessary for intron-dependent regulation of *CAS3* expression. **A**. Left panel. Northern blot experiment results showing the effect of *PAB2* deletion on *CAS3* mRNA accumulation. Central panel. Quantification of *CAS3* mRNA accumulation in the different genetic backgrounds and normalized to *ACT1* mRNA levels. These quantifications were done using RNA extracted from intact cells (grey bars) or from nuclei enriched fractions (white bars). The reported values are the means ± SD of three independent experiments. Right panel. Complementation of the capsule structure phenotypes as measured using the peroxidase-linked anti-capsule Mab 302. **B**. Left panel. Purification of a recombinant His-tagged-Pab2p in *E. coli*. 10% SDS/PAGE gel stained with Coomassie blue of *E. coli* lysate soluble supernatant (lane S), purified protein after affinity Column purification (lane E). Central panel. Pab2p binding assays. Affinity of a recombinant His-tagged Pab2p produced in *E. coli* to poly(A) was tested in a 96 well-plate format. Each well was coated with a poly(A) 30-mer oligonucleotide RNA. After incubation and washes, the quantity of bound proteins was estimated using an anti-His peroxidase linked monoclonal antibody. The affinity of Pab2p to poly(A) RNA and to poly(A) DNA were compared. Right panel. Competition assays in which 10 µM of unlabeled poly(A) or poly(C) (RNA) were added to the protein solution. **C**. Cells expressing the GFP-tagged version of the Pab2 protein were grown on glucose at 30°C and examined by epifluorescence or under bright field. Hoechst staining was used as control.

A *pab2Δ cas3Δi* strain was constructed by selecting adapted progenies after crossing the single mutant strains. Analysis of the expression of this intronless allele in a *pab2Δ* genetic background confirmed that this protein regulates *cas3Δi* expression. Indeed, as shown in Figure 4A, whereas *PAB2* deletion did not increase the expression of the *CAS3* wild type allele, it restored the expression of the intronless allele *cas3Δi* up to 12% of the wild type (Figure 4A, central panel, grey bars). We also confirmed by ELISA using another anti-capsule monoclonal antibody (Mab 302) that the level of mRNA accumulation in these strains correlated with the phenotype of the corresponding strains (Figure 4A, right panel).

We noticed the presence of two additional bands present in Northern blots for the *pab2Δ cas3Δi* mutant strain (Figure 4A, left panel, lane 4). These bands are probably products of partial degradation of the transcript or the result of partial transcription of the gene. Indeed, hybridizing with oriented RNA probes or with probes specific for the 5′ or 3′ends of the gene demonstrated that these additional bands correspond to the 5′end of the sense transcripts (Figure S4).

We also purified RNA from nuclei isolated using a similar protocol as the one used for the run-on experiments (see Material and Methods) and compared the accumulation of RNA obtained with this nuclei enriched fraction with the ones obtained with RNA extracted from intact cells. As shown in Figure 4A (middle panel), we observed a more pronounced accumulation of the *cas3Δi* mRNA in the nuclei enriched fraction (white bars), this more pronounced accumulation being exacerbated by a *pab2Δ* mutation. In good agreement with the localisation experiment data (see below), these results also suggested a nuclear role for Pab2p in the control of intronless allele expression.

Finally, we checked that Pab2p could also modulate the expression of intronless alleles of other genes by constructing a *pab2Δ cap10Δi* double mutant strain. As shown in Figure S5, the *PAB2* deletion also restored the *cap10Δi* mRNA level close to the wild type level confirming the role of Pab2p in the control of intronless allele expression.

### *PAB2* encodes a nuclear poly (A) binding protein

To functionally characterise the biophysical properties of Pab2p, we expressed an N-terminal 6XHis-tagged version in *E. coli* and purified the recombinant protein (see Material and Methods). We tested the affinity of this recombinant Pab2p towards a 30-mer poly(A) RNA coated in a 96-well plate well (see Material and Methods). We found that Pab2p recognized poly(A) oligonucleotides in a dose dependent way (data not shown) and that this recognition was specific to RNA as the same protein presented very little affinity to a 30-mer poly(A) DNA (Figure 4B). Similarly to what has been observed in *S. pombe* [48], competition assays suggested also that the binding was specific to poly(A) sequences as a poly(C) RNA sequence was not able to compete the binding of Pab2p to poly(A) (Figure 4B).

Next we constructed a *GFP::PAB2* allele to localize Pab2p within the cell. We transformed a *pab2Δ cas3Δi* strain and checked that the transformant selected grew as well as the wild-type strain at all temperatures tested. The functionality of the GFP::Pab2p fusion was confirmed by Northern blot which demonstrated that the fusion protein decreased expression of the allele *cas3Δi* (data not shown). Examination, by fluorescence microscopy showed a pattern of Pab2p of fluorescence consistent with nuclear localisation (Figure 4C). These results suggested strongly that Pab2p is a nuclear poly(A) binding protein.

### The exosome controls *cas3Δi* expression

Pab2p has been recently shown to interact with the two nucleases of the exosome (i.e. Rrp44p and Rrp6p) to control the synthesis of snoRNAs and the expression of meiotic genes [50]–[52]. It was thus very tempting to hypothesize that this multi-protein complex could regulate the expression of *cas3Δi* by degrading the RNA transcribed from this allele.

**Figure 5:**
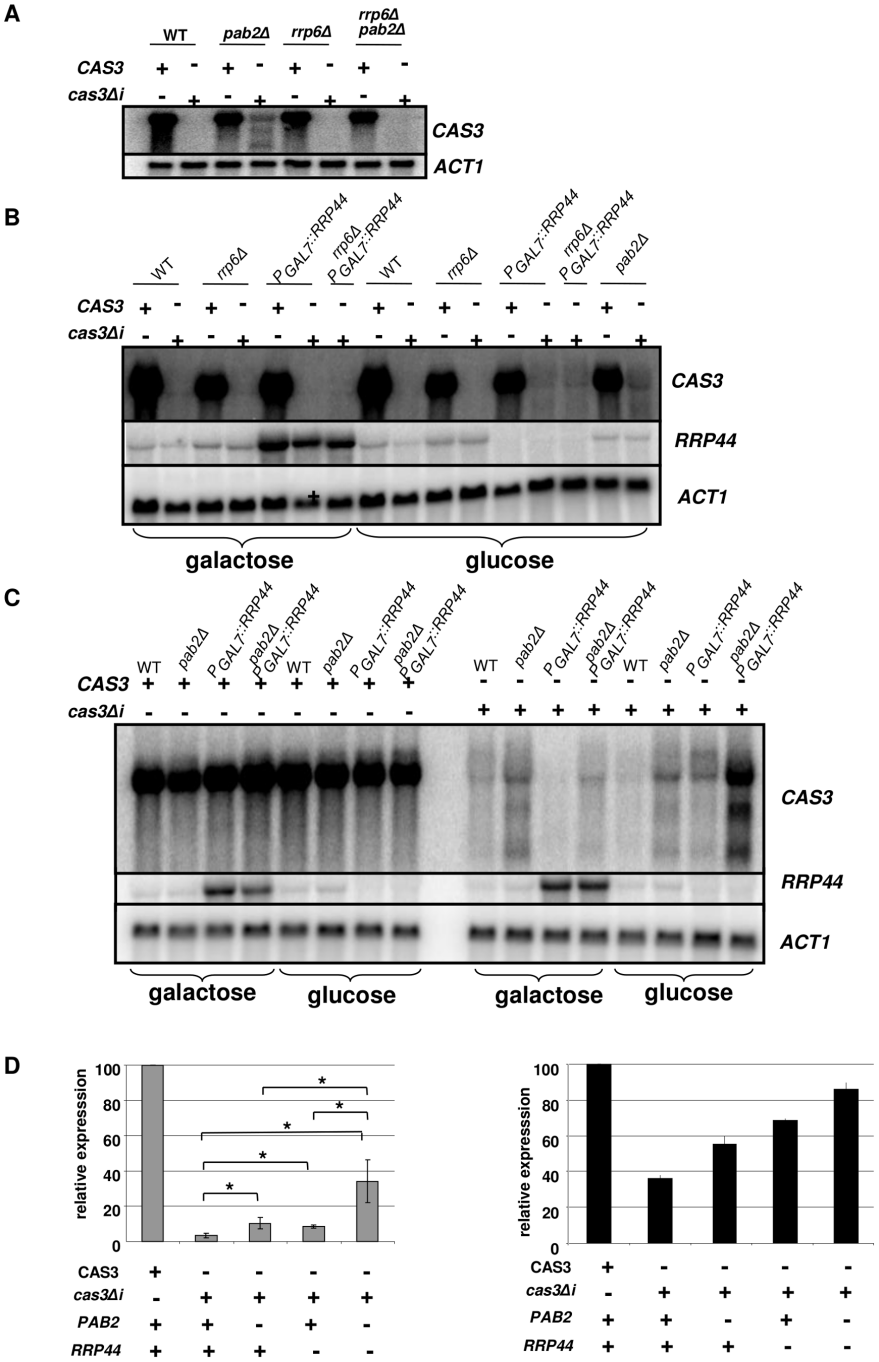
Regulation of *cas3Δi* mRNA accumulation by the exosome. **A**. Rrp6p does not regulate *cas3Δi* mRNA accumulation. RNA was extracted from cells growing in YPD (5·10^7^ cells/mL) and analyzed by Northern blot. **B**. Rrp44p regulates *cas3Δi* mRNA accumulation. Cells from different mutant strains grown overnight in galactose were washed and transferred to glucose for 6 h before RNA extraction. **C**. Synergic effect of *rrp44* and *pab2Δ* mutations. Cells from different mutant strains grown overnight in galactose were washed and transferred to glucose for 10 h before RNA extraction **D**. Left panel. Quantification of *CAS3* mRNA accumulation in the different genetic backgrounds. The measurements were normalized to *ACT1* mRNA levels. The reported values are the means ± SD of three independent experiments. (* means p<0.05 after student test). Right panel. Complementation of the capsule structure phenotype as measured using the peroxidase-linked anti-capsule Mab 302.

We identified the *RRP6* (gene CNC03940) and the *RRP44* (gene CND00800) homologues in the genome of *C. neoformans* and constructed corresponding mutant strains. As in the model yeasts *S. cerevisiae* and *S. pombe*, *RRP6* was not essential and we were able to delete this gene in *C. neoformans*. The phenotypes associated with the *RRP6* deletion were compared with the ones associated with the *PAB2* deletion. As presented in Figures 3A and 3B, *pab2Δ* and *rrp6Δ* strains presented a similar growth defect at 30°C. However, in contrast to what we observed with the *pab2Δ* mutant strains, the *rrp6Δ* mutants growth defect is not exacerbated when the cells are incubated at 15°C or 37°C and no hyper-sensitivity to SDS 0.01% was observed. The size and structure of the capsule and the urease production were not affected by the deletion of the *RRP6* gene (not shown). Interestingly, we observed the same slight defect in melanin production in *pab2Δ* and *rrp6Δ* mutant strains when the cells were grown on Niger medium (Figure 3B). Successive unsuccessful attempts to delete *RRP44* suggested that this gene is essential in *C. neoformans* as it is in *S. cerevisiae* and *S. pombe* [53], [54]. We thus expressed this gene under the control of the *GAL7* promoter which has been shown to be strictly regulated by the presence of galactose in the medium and can be used as a regulatable promoter in promoter swap experiments [55]. On galactose, these cells displayed no specific phenotype although *RRP44* was clearly over-expressed (Figure 5B and 5C) whereas on glucose, the *P_GAL7_::RRP44* strains failed to grow, confirming that this gene is essential in *C. neoformans* (Figure 3C).

To identify the nuclease that regulates expression of intronless *CAS3*, the *cas3Δi* allele was next introduced into the *rrp6Δ* and *rrp44* mutant strains. Moreover, we constructed all possible double mutant strains (*rrp6Δ pab2Δ*, *rrp6Δ P_GAL7_::RRP44* and *pab2Δ P_GAL7_::RRP44*) and we introduced the *cas3Δi* allele in all of them. Deletion of *RRP6* did not restore even partially the expression of *cas3Δi* (Figure 5A), suggesting that Rrp6p is not the nuclease degrading the RNA transcribed from the *cas3Δi* allele. Surprisingly, *RRP6* deletion in a *pab2Δ* background resulted in reversion to a complete absence of expression of the *cas3Δi* allele.

We next tested the influence of Rrp44p on the control of *cas3Δi* expression. To do so, we grew the different strains under the non-restrictive condition (galactose) and then transferred them to the restrictive condition (glucose). Preliminary experiments had shown that as early as 2 hours after the transfer of the cells to glucose medium no *RRP44* specific mRNA could be detected by Northern blot analysis when this gene was expressed under the control of the *GAL7* promoter (not shown). We compared mRNA levels of *CAS3* and *cas3Δi* under non-restrictive conditions and after 10 hours under restrictive growth conditions in the different genetic backgrounds. Whereas *CAS3* mRNA levels were similar in all mutant strains tested, incubation of *P_GAL7_::RRP44* cells under restrictive conditions (glucose) restored the expression of the intronless allele *cas3Δi* up to 9% of the wild type (Figures 5B and 5D). Similar results were obtained after shorter (6 h) incubation times. This mRNA level was not increased in the *rrp6Δ P_GAL7_::RRP44* double mutant confirming that Rrp6p is not implicated in this regulation. Interestingly, *RRP44* appeared to be up-regulated in the absence of *RRP6* suggesting a potential explanation for the absence of *cas3Δi* mRNA in the *rrp6Δ pab2Δ* double mutant (Figure 5A). The analysis of the double mutant *pab2Δ P_GAL7_::RRP44* revealed a synergic effect of these mutations. As shown in Figures 5C and 5D, the double mutant strains expressed the intronless allele up to 34% of the wild type. Accordingly, the level of mRNA correlated with the phenotypes of the corresponding strains (Figure 5D). These results strongly suggested that Rrp44p and thus the exosome participates in the degradation of mRNA transcribed from the intronless allele *cas3Δi*. They also demonstrated that the exosome is acting mainly independently of Pab2p suggesting the existence of a least two pathways regulating intronless expression in *C. neoformans*.

Several RNA species including snRNA, snoRNA, tRNA and rRNA are targeted to degradation by the exosome following polyadenylation by the TRAMP complex [56]. We thus addressed whether this nuclear complex has a role in the regulation of *cas3Δi* expression. Cid14p has been shown to represent the catalytic subunit responsible for the TRAMP complex poly(A) polymerase activity in *S. pombe* [57]. We deleted the single homologous gene (CNK02250) in the *C. neoformans* genome. Neither the *cid14Δ* strains nor the *cid14Δ pab2Δ* double mutant strains had any growth phenotype at any temperature tested (30°C, 37°C, 15°C) (not shown). Moreover, no alteration of *CAS3* or *cas3Δi* mRNA levels could be observed (Figure S6). Thus, Cid14p and the TRAMP complex do not seem to be implicated in the regulation of the expression of intronless alleles in *C. neoformans*.

Finally, as Pab2p has been previously implicated in poly(A) tail length control [58], we performed poly(A)-tests to examine the length of the poly(A) tail in the wild type or in the absence of Pab2p or Cid14p (see Material and Methods). The consequences of these gene deletions on the poly(A) tail length of 10 *C. neoformans* genes including *CAS3* were tested. However, we observed no reproducible modification of length of the poly(A) tails in any of the mutants tested (Figure S7, data not shown).

### Depletion of *XRN2* is compensated by *RRP44* and *vice versa*

In keeping with a primarily nuclear degradation of *cas3Δi* (as supported by the nuclear localisation of Pab2p and an enrichment in nuclear fractions) and given the results obtained for the two exosomal nucleases (Rrp6, Rrp44p), the major nuclear 5′→3′ exonuclease Xrn2p/Rat1p appeared to be the most promising candidate for Pab2p-assisted degradation of intronless mRNA. This idea was further encouraged by the finding that deletion of *PAB2* not only stabilises full-length *cas3Δi* but also truncated fragments corresponding to the 5′ end of the sense transcript thus suggesting the involvement of a 5′→3′ exonuclease. In addition, Xrn2p and Rat1p have been shown to be involved in the degradation of unspliced transcripts in human and yeast [59], [60].

**Figure 6:**
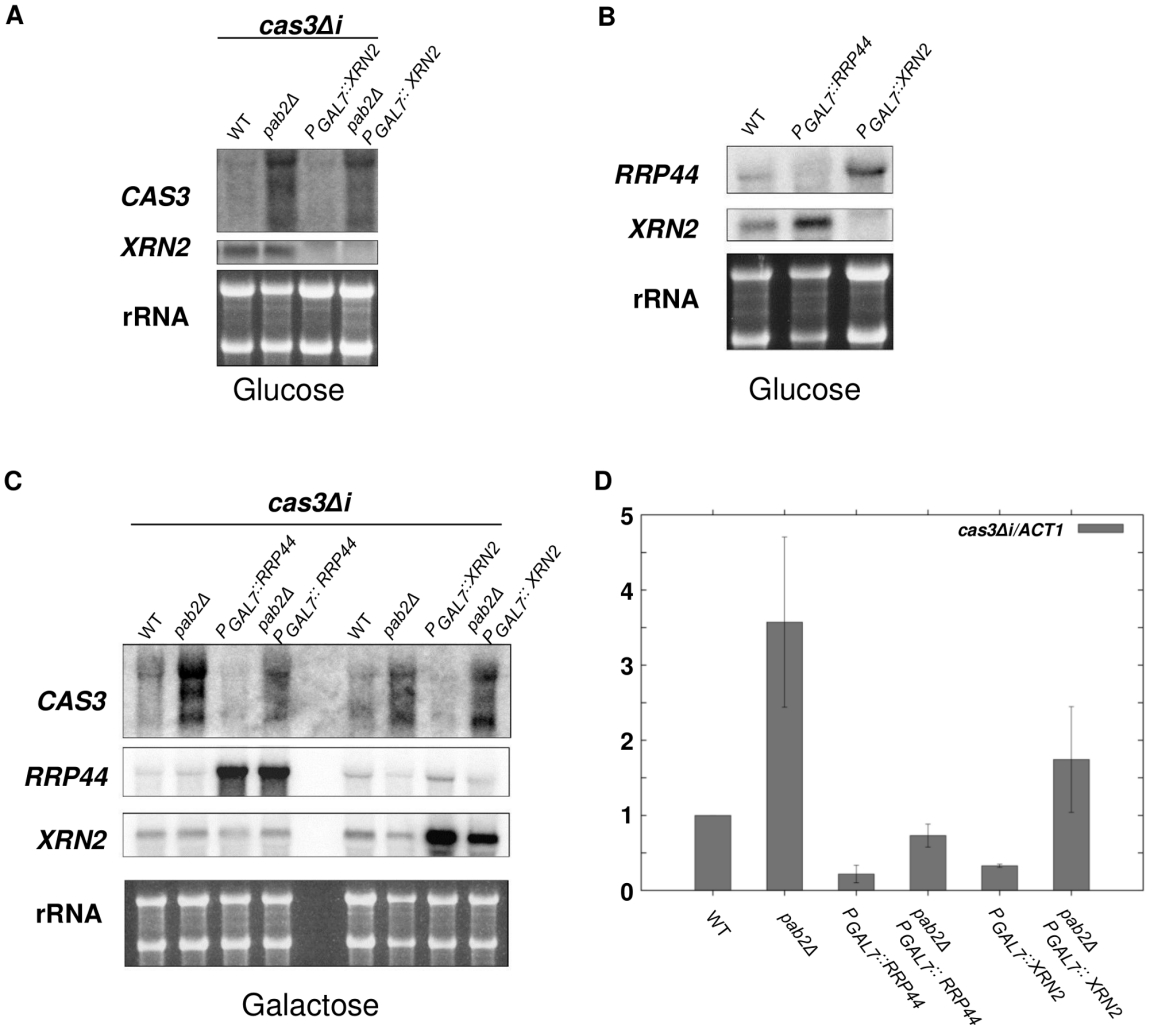
Xrn2p degrades *cas3Δi* mRNA in a Pab2p-dependent fashion. **A**. Simple depletion of *XRN2* does not lead to *cas3Δi* mRNA stabilisation. Cells from different mutant strains grown overnight in galactose were washed and transferred to glucose for 10 h. RNA was extracted and 5 µg were loaded on a denaturing electrophoresis agarose gel, transferred on a nylon membrane and probed with *CAS3* and *XRN2* specific probes. **B**. Depletion of *XRN2* leads to increased expression of *RRP44* and *vice versa*. Cells from different mutant strains grown overnight in galactose were washed and transferred to glucose for 10 h. RNA was extracted and 5 µg were loaded on a denaturing electrophoresis agarose gel, transferred on a nylon membrane and probed with *RRP44* and *XRN2* specific probes. **C. and D**. The degradation of *cas3Δi* mRNA by Rrp44p is largely independent of Pab2p whereas that by Xrn2p rather depends on Pab2p. RNA was extracted from cells growing in YPG (5·10^7^ cells/mL). 5 µg were loaded on a denaturing electrophoresis agarose gel, transferred on a nylon membrane and probed with *CAS3*, *RRP44* and *XRN2* specific probes C. RNA was treated with *Dnase* **I** and 1 µg were used to synthesise cDNA. Each quantitative PCR run was assayed in triplicate. For qPCR analyses primers were chosen that amplify 131 bp of the 5′ part of the *cas3Δi* transcript ensuring the capture of all isoforms of *cas3Δi* mRNA stabilised upon *PAB2* deletion. The reported values are the means ± SD of three independent experiments.

Given that *XRN2* is likely essential in *C. neoformans*, we placed the gene (CNF01810) under the control of the *GAL7* promoter, similar to the strategy applied for *RRP44*. As expected, cells failed to grow under restrictive conditions (glucose) showing that *XRN2* is indeed essential for *C. neoformans* viability. However, depletion of *XRN2* did not lead to any stabilisation of the *cas3Δi* transcript neither in a wildtype nor in a *pab2Δ* context (Figure 6A). Instead a slight decrease of *cas3Δi* mRNA was observed upon *XRN2* depletion in *pab2Δ* strains (Figure 6A). Accordingly, when we compared the levels of *RRP44* expression in wildtype and *xrn2* mutant cells, we found an increase in *RRP44* expression upon *XRN2* depletion (Figure 6B). Likewise expression of *XRN2* is elevated in cells depleted for *RRP44* (Figure 6B), which might partially explain the rather minor stabilisation of *cas3Δi* transcripts in *rrp44* cells.

To circumvent this compensatory effect and thus be able to evaluate the role of Xrn2p, we compared *cas3Δi* mRNA levels when overexpressing either *RRP44* or *XRN2* in wildtype and *pab2Δ* backgrounds. To this end, wildtype, *pab2Δ*, *rrp44*, *xrn2*, *pab2Δ rrp44* and *pab2Δ xrn2* strains were grown in inducing conditions (galactose) and the levels of *cas3Δi* expression were measured by Northern analysis. Note that upregulation of the intronless allele was reproducibly observed when the strains were grown in galactose (see Figure 5C). Overexpression of *RRP44* or *XRN2* in a *PAB2* wildtype context led to the nearly complete degradation of *cas3Δi* mRNA preserved by growth in galactose suggesting that both nucleases are implicated in the degradation of these mRNA molecules (Figure 6C). On the other hand, overexpression of *RRP6* did not lead to any destabilisation of the *cas3Δi* transcript thus confirming that Rrp6p has no central role in this regulation (data not shown).

In the absence of Pab2p, *RRP44* overexpression led to a strong decrease of *cas3Δi* mRNA accumulation and to nearly complete elimination of the two additional bands observed by Northern blot in a *pab2Δ* single mutant context (Figure 6C). In contrast, *XRN2* overexpression in a *pab2Δ* strain led to a very moderate or no decrease of *cas3Δi* mRNA accumulation. These results were confirmed by RT-qPCR (Figure 6D) although the effect of Xrn2p is probably overestimated in this assay due to the choice of primers specific for the transcript's 5′ end. In conclusion, these overexpression experiments confirm on the one hand that the action of Rrp44p is mainly independent of Pab2p and on the other hand suggest a rather Pab2p-dependent role for Xrn2p in the degradation of the intronless mRNA.

## Discussion

The role of introns on gene expression has been the focus of a large number of studies during the last decades [4]. Most of these studies are coming either from mammals or plants or have been performed using the intron-poor micro-organism *S. cerevisiae* as a model. In these organisms, the replacement of a wild-type gene by an intronless allele generally has a modest effect on gene expression suggesting that introns are more a source of protein diversity or/and regulation of gene expression than a *sine qua non* condition for a gene to be expressed [8], [18], [21], [61]. In contrast, expression of some genes like the human *β-globin* or the plant ERECTA genes is highly dependent on the presence of introns [10], [15]. Most of the intron-dependent regulation occurs at a post-transcriptional level although the different steps necessary for the production of a mature mRNA, including transcription and splicing are mutually dependant. [62]. In these cases the presence of introns in a transcript can affect 3′end formation, mRNA export from the nucleus and mRNA stability [8], [63]. However, the pathway(s) by which mRNA molecules transcribed from intronless alleles are recognized and degraded remain unknown [64].

The intron density of the pathogenic yeast *C. neoformans* is probably the highest yet known for an organism having a completely annotated genome. In fact, a recent re-annotation of the *C. neoformans* var. *grubii* genome based on RNA-Seq data showed that 99% of the expressed genes have at least one intron (Janbon, unpublished data). Moreover, 11.5% and 4.1% of genes in *C. neoformans* have been shown to have 5′ and 3′ UTR introns, respectively [65]. It has also been previously published that alternative splicing is very common in *C. neoformans* [40], [42]. More recently, a link between transposon pre-mRNA splicing and RNAi dependent degradation has been demonstrated in *C. neoformans* [66]. Altogether, these data suggested a central role for intron metabolism in the biology and the virulence of *C. neoformans*. In this study, using the gene *CAS3* as a model transcript, we showed that alternative splicing can affect all introns from a single gene although their spliceability appeared to be intron-dependent. We also demonstrated that introns are necessary for the *CAS3* gene expression in *C. neoformans*. Three other tested genes have the same intron-dependence of gene expression whereas another one (*UXS1*) can be expressed without introns. This insensitivity to the lack of introns does not appear to depend on the number of introns. Indeed *CAP10* has only 3 introns, *UGE1* only 4 whereas *CAS4* and *UXS1* have 9 and 7 introns, respectively. Moreover, it probably does not depend on the presence of an intron in the UTR as *CAS3* is the only one of the presently studied genes to possess such an intron. It has to be noted that the fact that intronless bacterial antibiotic resistance genes are commonly used for mutant construction in *C. neoformans* does not contradict this observation. Indeed, all these genes are expressed under the control of the *ACT1* promoter, in which an intron is present [67]–[69].

Our results demonstrated that most introns play a positive role on mRNA accumulation and that the absence of introns does not alter the level of transcription as measured by run-on transcription assay. These results are similar to what has been observed in mammals, in the fungus *S. commune* or for IME in plants in which the regulation of gene expression by introns acts mainly at a post-transcriptional level [8], [31], [61]. In contrast to what has been observed in most cases however, one intron is not enough to restore gene expression. Even with two introns the mRNA level remained below 3% of the wild-type. Most introns played a positive role on gene expression and their action seemed to be more cumulative than specific as previously reported for the ERECTA gene in *A. thaliana* [15]. The two most external introns (1 and 12) played a negative role on *CAS3* mRNA accumulation. Run-on experiments suggested no transcription rate alteration associated with the deletion of either one of these introns (data not shown) suggesting also a post-transcriptional regulatory mechanism. More investigations are obviously needed to understand the role of these introns on mRNA accumulation.

The absence of introns results in an important reduction of mRNA accumulation. Deletion of the *PAB2* gene partially stabilized mRNA transcribed from the intronless allele. In contrast, the analysis of the *cid14Δ* strains suggests no apparent role for the TRAMP complex in this regulation [52]. Pab2p has been shown to interact physically with the two nucleases (Rrp6p and Rrp44p/Dis3p) from the exosome in *S. pombe* [50]. In *C. neoformans*, although the deletion of *RRP6* encoding the nuclear exosome nuclease has no effect on the accumulation of mRNA transcribed from *cas3Δi*, the analysis of the level of *cas3Δi* mRNA in a *RRP44* conditional mutant under a restrictive condition strongly implicates this multiprotein complex in this regulation. The analysis of the double mutant strain *pab2Δ rrp44* demonstrated a synergic effect of the two mutations suggesting that these two proteins could act in two independent pathways. [70], [71]. As suggested in other Pab2p dependent pathways described to date, Pab2p could be a facilitator for the degradation of *cas3Δi* mRNA through recruitment of another nuclease. Our double mutant strains analysis and overexpression experiments suggest that the nuclear 5′→3′ exonuclease Xrn2p might represent a good candidate. This model could explain the synergic effect of the *pab2Δ rrp44* double mutation. Thus, in the single *rrp44* mutant strain, *XRN2* is over expressed and can partially compensate the effect of Rrp44p depletion whereas in the absence of Pab2p, *XRN2* over-expression would have much less effect on the degradation of *cas3Δi* transcripts. The fact that Xrn2p/Rat1p has been previously shown to be involved in the degradation of unspliced mRNA in human and yeast [59], [60] sustains this model although no genetic interaction between Xrn2p and Pab2p has been reported to date.

Very recently, Pab2p has been shown to be involved in three different RNA processing and degradation pathways in *S. pombe*. Thus, together with the exosomal nucleases Rrp6p and independently of the TRAMP complex it controls polyadenylation and synthesis of snoRNAs [50], meiotic gene expression in the Mmi1-dependent pathway [51], [52], [72] and targets ribosomal pre-mRNA *RPL30-2* [73]. Similarly, in the meiotic gene expression and pre-mRNA RPL30-2 regulation, a synergic effect was observed when *RRP44* and *PAB2* were mutated suggesting here also that the effect of Rrp44p could be mainly independent of Pab2p although the other elements involved in this Rrp44p-dependent pathway remain to be identified.

**Figure 7:**
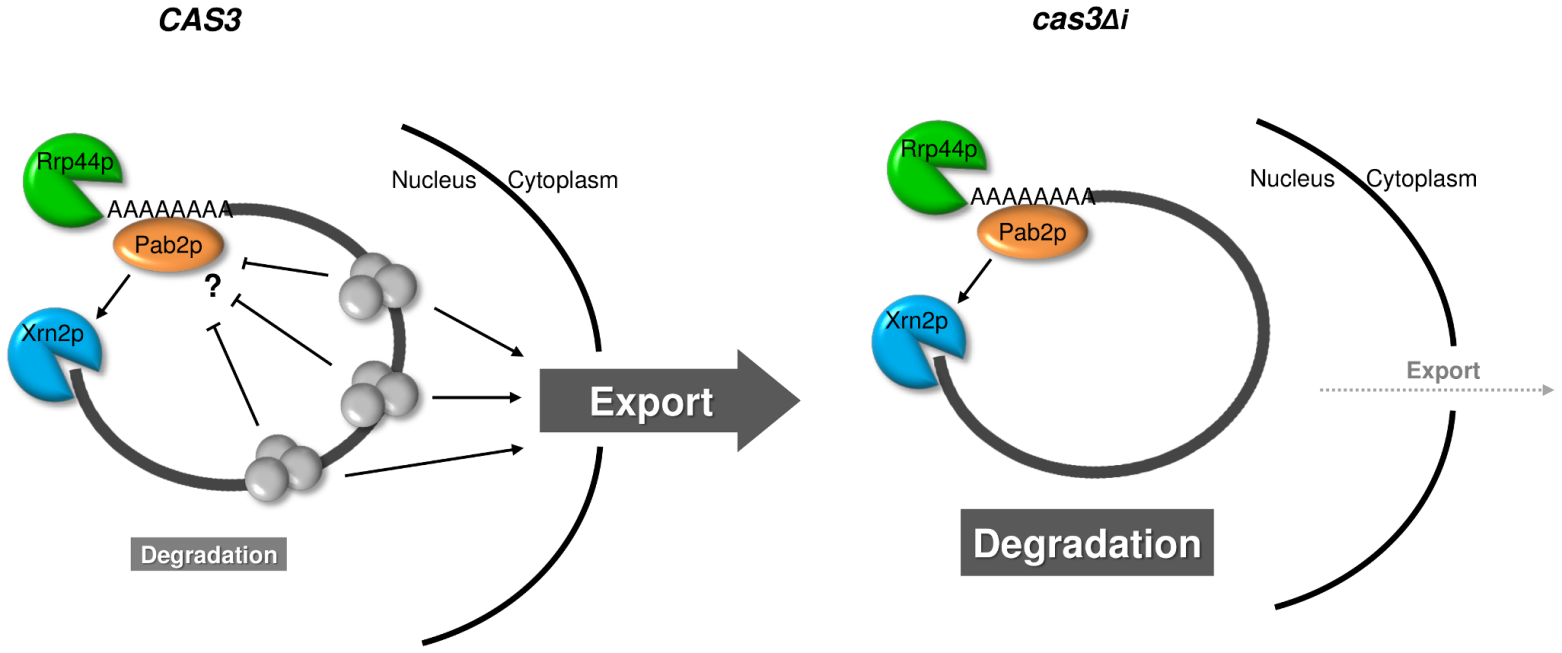
Model for intron-dependent gene expression regulation in *C. neoformans*. In a wild type context (*CAS3*), protein complexes (EJC) are deposited on the mRNA upon splicing and facilitate mRNA export. The presence of these complexes might also protect mRNA from degradation. In the intronless context (*cas3Δi*), the absence of splicing impedes EJC deposition and the mRNA is inefficiently exported giving more time to Rrp44p and Xrn2p/Pab2p to degrade it.

The role of Pab2p in the intronless gene expression regulation remains mysterious. In *S. pombe*, Pab2p is recruited to the nascent mRNA before 3′ end formation and polyadenylation and controls the length of poly(A) of only a subset of RNAs [48], [50], [71]. It also physically interacts both with the exosome nucleases and the poly(A) polymerase Pla1p [52], [71]. In the absence of Pab2p, some *cas3Δi* mRNA molecules are exported from the nucleus and translated although most of them are still degraded. The subcellular localisation of Pab2p together with the analysis of the accumulation of the mRNA transcribed from the intronless allele in the nucleus, suggested strongly a nuclear role for this protein although Pab2p has been shown to be able to shuttle to the cytoplasm in *S. pombe* and in *Drosophila* [70], [71]. The kinetic of degradation of intronless mRNA in *C. neoformans* might be the result of a disequilibrium between mRNA export from the nucleus and degradation. Thus, when not enough introns are present the altered dosage of mRNA binding proteins would result in an extended retention time of mRNA in the nucleus giving time to the nucleases to degrade them. The absence of Pab2p would slow down this degradation giving time to some mRNA molecules to be exported in a “take the money and run” strategy [74] (Figure 7).

In terms of evolution, the comparison of *S. cerevisiae* and *C. neoformans* provides a fascinating example of opposite evolutionary choices. Whereas *S. cerevisiae* has lost almost all its introns and has largely simplified its RNA metabolism (i.e. loss of RNAi pathway, only one SR protein, absence of EJC-like complex…), *C. neoformans* has conserved and maybe increased its intron number and appears to have a very complex RNA metabolism. The selective pressure that has maintained introns in one organism and has eliminated them in another one is unknown. It has to be noted that *C. neoformans* is not a unique example among basidiomycete fungi. Thus, genes from *Coprinus cinereus* and *Phanerochaete chrysosporium* have an intron density close to that of *C. neoformans* [30]. In two other pathogens, *Ustilago maydis* for the plants and *Malassezia sp.* for human for example, the number of genes with introns is small [75], [76]. This specificity might be related to the fact that C. *neoformans* is an opportunistic human pathogen living in the environment. As such the diversity of signals to which it can be exposed in the human body or in soil for example is huge. Indeed, this organism has to cope with a large number of different stresses and probably needs a very flexible metabolism. It is tempting to hypothesize that its complex RNA metabolism provides a mechanism to achieve such flexibility.

## Materials and Methods

### Strains and culture conditions

*C. neoformans* strains used in this study all originated from the serotype D strain JEC21 [77] and are listed in Table S1. The strains were routinely cultured on YPD medium at 30°C [78]. Synthetic dextrose (SD) was prepared as described [78]. The capsule sizes were estimated after 24 h of growth in capsule-inducing medium at 30°C as previously described [79]. Melanin and urease production were assessed after spotting 10^5^ cells of each strain on Niger or Christensen agar medium, respectively [69], [80]; the plates were read after 48 h of incubation at 30°C. The bacterial strain *Escherichia coli* XL1-blue (Stratagene) was used for the propagation of all plasmids.

### RNA extraction and Northern blot analysis

Cells were routinely harvested after being grown up to 5·10^7^ cells/mL in YPD. RNA was extracted with TRIZOL Reagent (Invitrogen) following the manufacturer's instructions. Total RNA (5 µg) was separated by denaturing agarose gel electrophoresis and transferred onto Hybond-N+ membrane (Amersham) and probed with [^32^P]dCTP-radiolabelled DNA fragments. The banding pattern was quantified with a Typhoon 9200 imager and Image Quantifier 5.2 software (Molecular dynamics).

### *CAS3* cDNA analysis

Total RNA was extracted from JEC21 cells growing on YPD. mRNA was purified using Oligotex Direct mRNA Mini Kit (Qiagen) following the manufacturer's instructions. SMARTer RACE cDNA Amplification Kit (Clontech) was used to synthesize the cDNA. *CAS3* cDNA was PCR amplified using the primers CAS3a and CAS3AR (see Table S2) and was analysed by agarose gel electrophoresis. The presence of a smeary pattern on the gel suggested the presence of different types of cDNA molecules. The amplified fragments were gel purified in three different pools of sizes and 15 cDNA molecules from each pool were cloned in a pGEMT plasmid and sequenced.

### Insertional mutagenesis

An insertional mutant library was constructed in a NE292 (*MAT* **a** *cas3Δi ura5*) background using the *Agrobacterium tumefaciens* strain EHA105 transformed with the plasmid pPZP-NEO1 as previously described [81]. A total of 5796 colonies were transferred from the transformation plates to 96-well plate wells containing 75 µL of capsule inducing medium [79] supplemented with adenine (20 mg/L) and uracil (5 mg/L). The mutants were then tested with the anti-capsule monoclonal antibody CRND-8 [82] as previously described [83]. Positive mutant strains were isolated and tested a second time using the same strategy. Total RNA was extracted and the presence of *CAS3* mRNA was analysed by Northern blot.

### Recombinant protein production

*PAB2* cDNAs were amplified by PCR and inserted into the pQ-30 *E. coli* expression vector (Qiagen). The *E. coli* BL21 transformant strains were grown in 50 mL of YT containing ampicillin (50 µg/ml) and kanamycin (30 µg/ml) to an OD_600_ of 0.5; gene expression was induced by adding 1 mM of IPTG and incubation for 4 hours at 37°C. The cells were then disrupted by sonication and centrifuged at 3000·*g*. The supernatant was recovered and the recombinant proteins were purified by affinity chromatography on a Ni-NTA column (Qiagen) following the manufacturer's procedures. The protein solution was adjusted to 20% (w/v) glycerol (final concentration 140 µg/mL) and stored in aliquots at −80°C.

### Poly(A) binding assays

These experiments were conducted in 96-well Streptavidin coated plates (Nunc). For each sample and concentration to be tested one well was washed three times with 200 µl of washing buffer (Tris 25 mM, Nacl 150 mM, pH 7.2, BSA 1% wt/vol, Tween 20 0.05% wt/vol). Each well was then incubated for 2 h at room temperature and under agitation (700 rpm) with 100 µl of washing buffer containing 0.1 µM of poly(A) 30-mers oligonucleotide 5′ biothinylated. Unbound oligonucleotides were then eliminated through three washes with 200 µL of washing buffer. Each well was then incubated with 100 µl of recombinant Pab2p solution (0.70 mg/mL) for 30 min at room temperature under agitation (700 rpm). After three washes with 200 µL of washing buffer, the quantity of poly(A)-bound protein was estimated using an anti-His peroxidase linked monoclonal antibody (Qiagen) and OPD (O-phenylenediamine dihydrochloride) (Sigma) following the manufacturer's procedures. After 10 min of incubation at room temperature, the colorimetric reaction was stopped by the addition of H_2_SO_4_ 4% (v/v) and the optic density was measured at 492 nm. For the competition assays, 10 µM of unlabeled poly(A) or poly(C) (Sigma) was added to the protein solution.

### Pab2p subcellular localization with fluorescent protein fusion

To localize the Pab2 protein, the *PAB2* gene under the control of its own promoter was joined in-frame to a sequence encoding the GFP protein at its N-terminal end. Primers used for amplification are listed in Table S2. A *pab2Δ* strain was transformed with a plasmid containing the *URA5* marker and the Pab2-fluorescent protein fusion by biolistic delivery [84]. Transformants were grown on minimum medium and analyzed for fluorescence.

### Intron allele construction

The pBluescript (Stratagene) based plasmid pNE247 contained a 4067 bp DNA fragment containing the complete *C. neoformans CAS3* gene PCR amplified using the primers CAS3F and CAS3R (see Table S2) and cloned at the *Not* **I** site. This plasmid was used to construct all the *CAS3* alleles presented in this study. For the intronless allele, the cDNA from *CAS3* was amplified, cloned and sequenced (see above). A completely spliced molecule was digested with *Sph* **I** and *Pst* **I** and cloned at the *Sph* **I**-*Pst* **I** site of pNE247, thus replacing the wild type gene by an intronless version under the control of its own promoter. 5 µg of the resulting plasmid pNE254 were *Not* **I** digested and mixed with 1 µg of the *URA5* containing plasmid pNE10 [79] digested with *Not* **I**. This DNA solution was used to transform the strain NE128 (*MAT* **a** *cas3Δ:ADE2 ura5*) [46] by biolistic transformation. The transformants were selected on a minimum medium containing adenine at 20 mg/L. After three days at 30°C, the transformation plates were transferred to room temperature and one week after transformation some colonies developed a pink phenotype suggesting that the *ADE2* gene had been lost and thus that the *cas3Δ::ADE2* allele has been replaced by the *cas3Δi* allele. The pink colonies were then cultured in liquid YPD so as to loose the unstable pNE10 plasmid [79]. Ura^−^ strains were selected on FOA. The absence of the *cas3Δ::ADE2* allele and the correct integration of the intronless allele were confirmed by PCR. The absence of additional integrations in the genome was confirmed by Southern blot. Two independent mutant strains were selected and stored at −80°C. Similar strategies were used to construct the other alleles and the other mutant strains.

### Nuclear run-on assay and nuclear fraction preparation

For nuclei purification, 500 mL of culture (OD_600_ = 3) were harvested by centrifugation. Spheroplasts were prepared as previously described [85] and re-suspended in 3 mL of lysing buffer (Pipes 10 mM pH 6.9, sucrose 0.5 M, CaCl_2_ 5 mM, MgSO_4_ 5 mM, DTT 1 mM) containing a complete set of antiproteases (Roche). The spheroplasts were then mechanically disrupted and the intracellular organelles were separated from cellular debris and unbroken cells by centrifugation. Nuclei were purified by differential ultracentifugation (1 hour, 161 000·g, 4°C) through a separation buffer (Pipes 10 mM, sucrose 2.1 M CaCl_2_ 5 mM, MgSO_4_ 5 mM, DTT 1 mM) containing a complete set of antiproteases (Roche). Nuclei were then washed twice with conservation buffer (TrisHCl 50 mM pH 8.3, glycerol 40%, MgCl_2_ 5 mM, EDTA 0.1 mM pH 8), re-suspended in 500 µl of conservation buffer and stored in aliquots at −80°C. For each run on experiment 100 µL of nuclei suspension was used following a protocol previously described [47]. The radioactive transcripts produced were used to hybridize a serial dilution of DNA spotted on a nylon membrane. The plasmid pNE428 containing the 1558 bp fully spliced JEC21 *CAS3* cDNA amplified with the primers CAS3a and CAS3AR and cloned in the pGEMT plasmid (Clontech) was used as *CAS3* specific DNA. The plasmid pNE435 containing the DNA 519 bp DNA fragment PCR from JEC21 genomic DNA using the primers ACT1F and ACT1R cloned in pGEMT was used as *ACT1* specific DNA. The plasmid pGEMT alone was used as negative control. The intensity of the signal was quantified with a Typhoon 9200 imager and Image Quantifier 5.2 software (Molecular dynamics). Each experiment was repeated twice using two independent nuclei preparations. The same protocol of nuclei preparation was used to isolate the nuclear RNA fraction. Electrophoretic analysis of these RNA samples showed a clear decrease in the rRNA proportion confirming the quality of our preparation (data not shown).

### Gene disruption

The genes described in this report have been deleted by biolistic transforming a serotype D strain using a disruption cassette constructed by overlapping PCR as previously described [45]. The primer sequences used are given in Table S2. The transformants were then screened for homologous integration as previously described [46]. The plasmid, pNAT used to amplify the NAT selective marker was kindly provided by Dr Jennifer Lodge (Saint Louis University School of Medicine). The plasmid pPZP-NEO1 used to amplify the NEO selective marker was kindly provided by Dr Joe Heitman (Duke University). Multiple mutant strains were obtained through crosses of single mutant strains on V8 medium as previously described [45]. Progenies were selected on minimum medium to which different amino acids were added. Their genotypes were determined by PCR. The mating types of the strains were determined by testing them on V8 medium in the presence of tester strains of known mating type.

### Promoter swap

A four way overlap PCR gene deletion was used to generate the promoter-specific exchange cassettes of *RRP44 and XRN2*, which included a nourseothricin and a neomycin cassette, respectively. The primers used in these experiments are listed in Table S2. The *GAL7* promoter was used as the inducible promoter [55]. The 694 bp upstream the *RRP44* ATG and the 601 bp upstream the *XRN2* ATG were replaced by the 556 bp present upstream of the *GAL7* gene. Transformants were screened for homologous integration as previously described [86].

### Measure of polyadenylation

The ePAT and TVN-PAT reactions were performed using 1 µg of input RNA as previously described [87]. The sequences of the primers used for PCR amplification are listed in Table S2. The cDNA was column-purified using NucleoSpin Gel and PCR Clean-up columns (MACHEREY-NAGEL). Specific PCR products were analysed by 2% high resolution agarose gel (Ultra pure 1000; Life Technologies) pre-stained with sybr safe (Life Technologies) and imaged against a 100 bp ladder (New England Biolabs) using an LAS 3000 imager and multigauge software (Fujifilm).

### RT-qPCR

Total RNA was subjected to an initial *DNase* **I** (Roche) treatment to eliminate contaminating genomic DNA. 1 µg of the *DNase* **I** treated RNA was then reverse transcribed using the kit QuantiTect Reverse Transcription (Qiagen) following the manufacturer's instructions.

Quantitative PCR assays were performed according to Bio-Rad manufacturer's instructions using 96-well optical plates (Thermo Scientific) and an iCycler iQ (170–8740, Biorad).

Each run was assayed in triplicate in a total volume of 25 µL containing the 5 µL cDNA template at an appropriate dilution, 1× Absolute qPCR SYBR Green Fluorescein (Thermo Scientific) and 320 nM of each primer. The primers used are listed in Table S2. PCR conditions were: 95°C/15 min for one cycle, 95°C/30 s for 40 cycles. Amplification of one single specific target DNA was checked with a melting curve analysis (+0.5°C ramping for 10 s from 55°C to 95°C). The Ct values obtained in triplicate were averaged and normalised to that of the housekeeping gene *ACT1* using standard curves. To verify the absence of genomic DNA contamination, negative controls in which reverse transcriptase was omitted were used. Three independent biological replicates were performed.

## Supporting Information

Figure S1 Typical results obtained after Run On experiment. Serial dilution of *CAS3* and *ACT1* specific DNA (see Material and Methods) were spotted on Nylon membrane and probed with radioactive transcripts.
(TIF)

Figure S2 Swapping the intron 12 and 2 does not alter mRNA accumulation. The intron 2 (red box) was placed at the intron 12 (blue box) position (strain NE806) and reciprocally. The mRNA levels of the different strains were estimated after Northern analysis and normalized to *ACT1* gene mRNA accumulation.
(TIF)

Figure S3 Phenotypic analysis of the strains bearing the different *CAS3* alleles. Serial 2-fold dilutions of cell suspensions were spotted (starting with 3·10^4^ cells on the first spot at the top of the lane) on a nitrocellulose membrane and probed with the anti-capsule Mab CRND-8.
(TIF)

Figure S4 Hybridisations with strand- or portion-specific probes. Top panel. Positions and orientations of the different probes used in this study. The sense (Probe E) and antisense (Probe D) probes were RNA probes synthesized using the same DNA substrate as the one used for the probe A. Bottom panel: Results obtained with the different probes after Northern hybridization.
(TIF)

Figure S5 Suppression of the intron-dependent *CAP10* gene expression by a *pab2Δ* mutation. RNA was extracted from cells growing YPD (5·10^7^ cells/mL) and 5 µg were loaded on a denaturing electrophoresis agarose gel, transferred on a nylon membrane and probed with *CAP10* and *ACT1* specific probes.
(TIF)

Figure S6 *CID14* does not regulate *cas3Δi* expression. RNA was extracted from cells growing on YPD (5·10^7^ cells/mL) and 5 µg were loaded on a denaturing electrophoresis agarose gel, transferred on a nylon membrane and probed with *CID14* and *ACT1* specific probes.
(TIF)

Figure S7 *PAB2* and *CID14* do not regulate poly(A) tail length. **A**. The ePAT and TVN-PAT reactions generate cDNA that include either the full poly(A)-tail or an invariant short (A-12) poly(A) tail, respectively as indicated by the reverse (R) primers. The position of the forward (F) gene-specific primer dictates the size and complexity of the amplified product. Thus, in the case of *CAS3*, in which the 3′ UTR intron (intron 12, black bar) is often retained, the CAS3 (F1) primer amplifies cDNA from both the spliced and unspliced transcript. The two 3′ RACE forms manifest as either tight bands of fixed-size PCR amplicons, or smears of amplicons, reflecting the steady-state distribution of poly(A)-tails. **B**. ePAT and TVN-PAT cDNA generated from the indicated strains were subject to 28 cycles of PCR amplification with the indicated primers. The CAS3 (F1) primer picks up both the spliced and intron-retained (A-12*) isoforms of *CAS3*. The numbers A-12, A-50, A-100 etc. refer to the length of the poly(A)- tail included in PCR amplicons at the given positions in the gel. To better compare the lengths of the poly(A) tail associated with *CAS3* in the various strains, the CAS3 (F2) splice junction spanning primer was used. The *CAP10* transcript is included as an assay and loading control.
(TIF)

Table S1 List of the *C. neoformans var neoformans* strains used in this study.
(DOC)

Table S2 List of the primers used in this study.
(DOC)
