# Supplementary figures and images for: Introns Regulate Gene Expression in Cryptococcus neoformans in a Pab2p Dependent Pathway
Source: PLoS Genet. 2013 Aug 15;9(8):e1003686. doi: 10.1371/journal.pgen.1003686 (PMC3744415; doi:10.1371/journal.pgen.1003686)

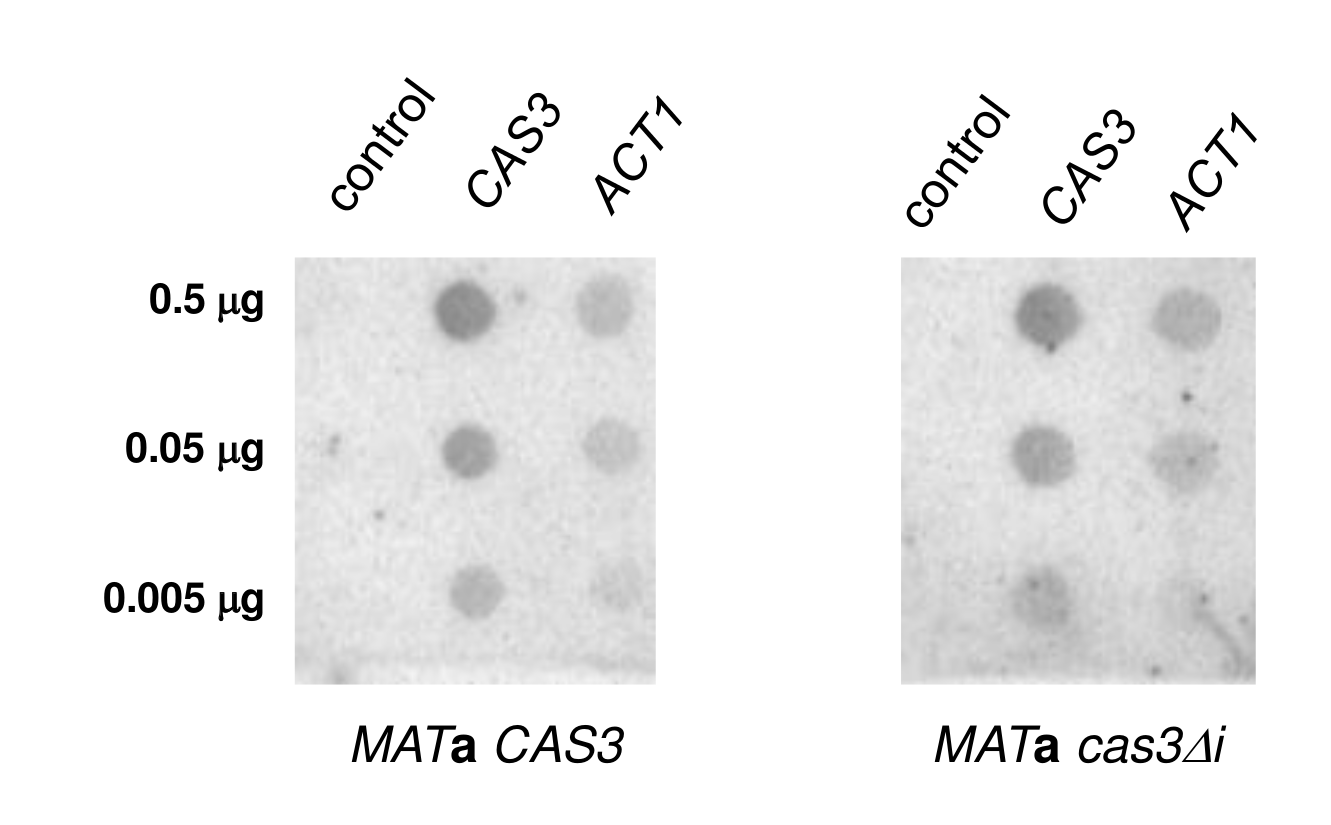

Supplement: Figure S1 — Typical results obtained after Run On experiment. Serial dilution of CAS3 and ACT1 specific DNA (see Material and Methods) were spotted on Nylon membrane and probed with radioactive transcripts. (TIF) [file pgen.1003686.s001.tif]

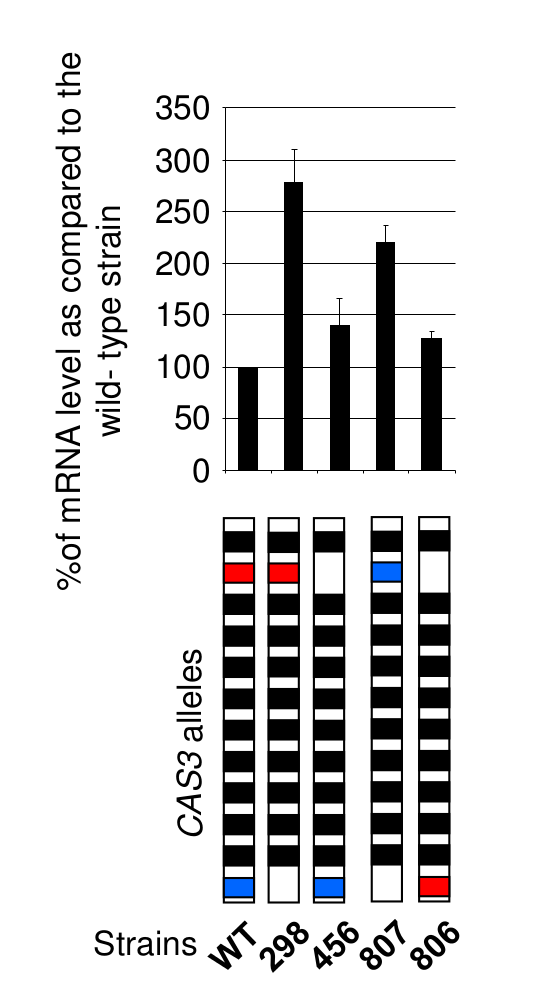

Supplement: Figure S2 — Swapping the intron 12 and 2 does not alter mRNA accumulation. The intron 2 (red box) was placed at the intron 12 (blue box) position (strain NE806) and reciprocally. The mRNA levels of the different strains were estimated after Northern analysis and normalized to ACT1 gene mRNA accumulation. (TIF) [file pgen.1003686.s002.tif]

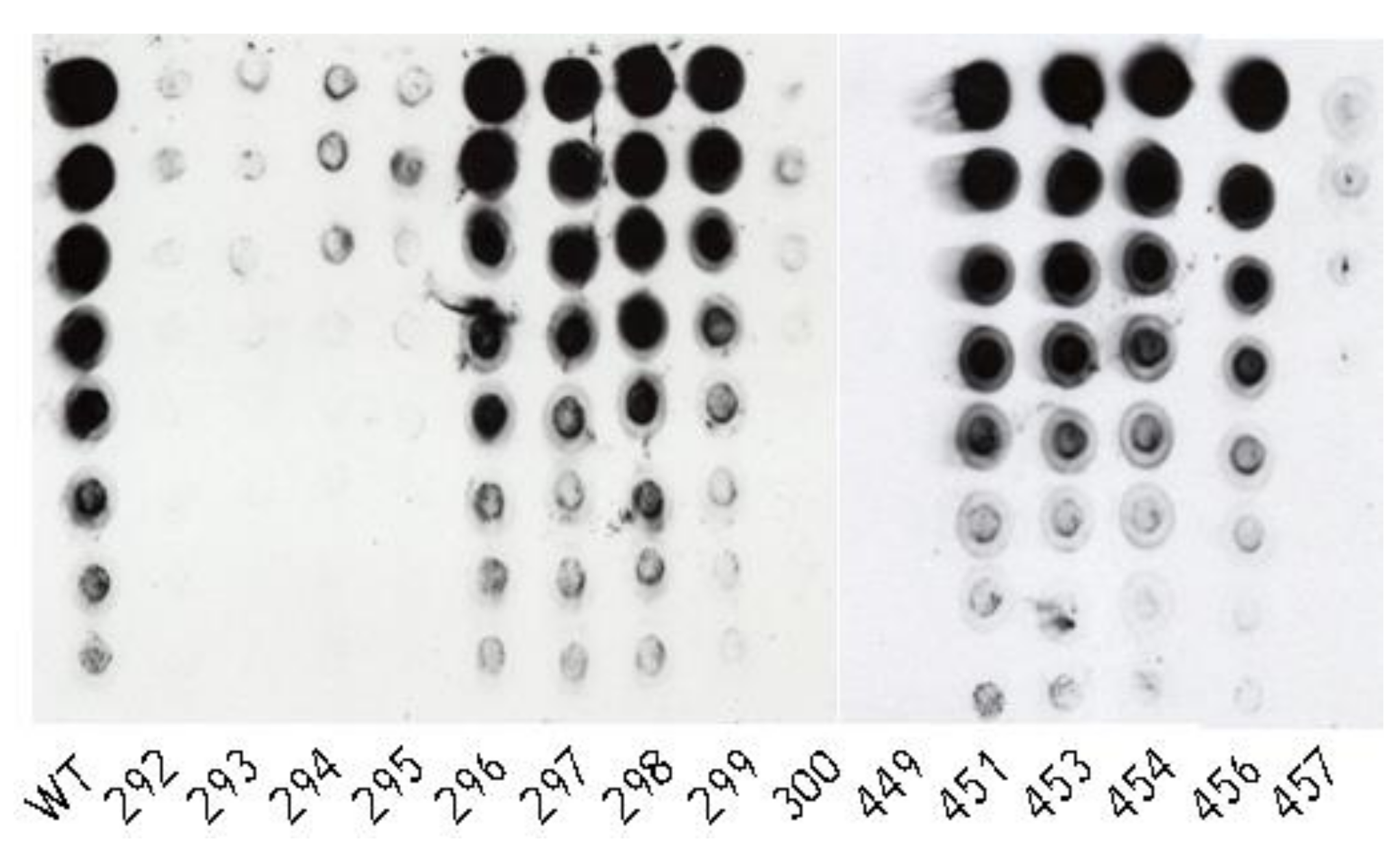

Supplement: Figure S3 — Phenotypic analysis of the strains bearing the different CAS3 alleles. Serial 2-fold dilutions of cell suspensions were spotted (starting with 3·104 cells on the first spot at the top of the lane) on a nitrocellulose membrane and probed with the anti-capsule Mab CRND-8. (TIF) [file pgen.1003686.s003.tif]

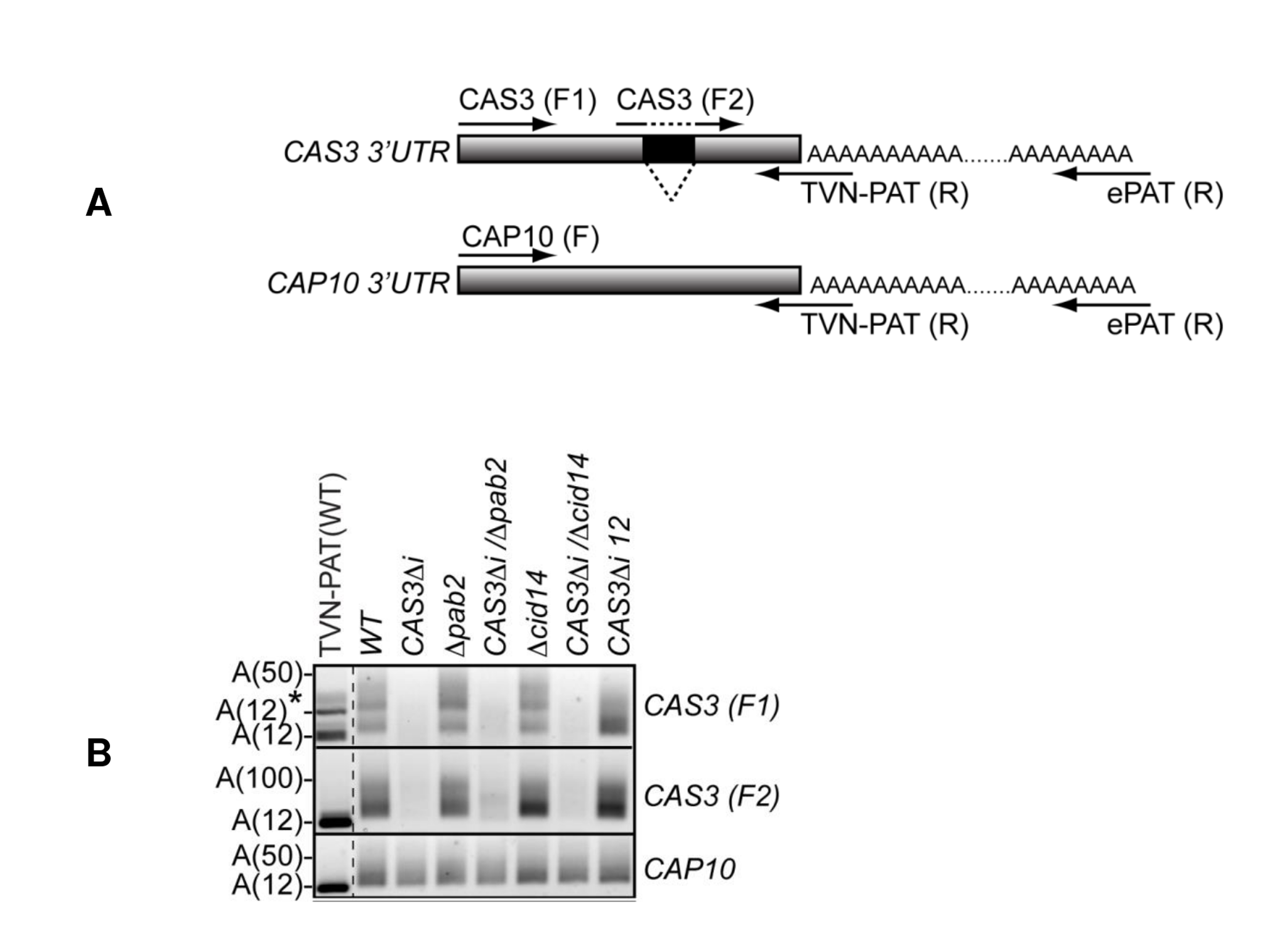

Supplement: Figure S7 — PAB2 and CID14 do not regulate poly(A) tail length. A. The ePAT and TVN-PAT reactions generate cDNA that include either the full poly(A)-tail or an invariant short (A-12) poly(A) tail, respectively as indicated by the reverse (R) primers. The position of the forward (F) gene-specific primer dictates the size and complexity of the amplified product. Thus, in the case of CAS3, in which the 3′ UTR intron (intron 12, black bar) is often retained, the CAS3 (F1) primer amplifies cDNA from both the spliced and unspliced transcript. The two 3′ RACE forms manifest as either tight bands of fixed-size PCR amplicons, or smears of amplicons, reflecting the steady-state distribution of poly(A)-tails. B. ePAT and TVN-PAT cDNA generated from the indicated strains were subject to 28 cycles of PCR amplification with the indicated primers. The CAS3 (F1) primer picks up both the spliced and intron-retained (A-12*) isoforms of CAS3. The numbers A-12, A-50, A-100 etc. refer to the length of the poly(A)- tail included in PCR amplicons at the given positions in the gel. To better compare the lengths of the poly(A) tail associated with CAS3 in the various strains, the CAS3 (F2) splice junction spanning primer was used. The CAP10 transcript is included as an assay and loading control. (TIF) [file pgen.1003686.s007.tif]
