# Supplementary material for: Introns Regulate Gene Expression in Cryptococcus neoformans in a Pab2p Dependent Pathway
Source: PLoS Genet. 2013 Aug 15;9(8):e1003686. doi: 10.1371/journal.pgen.1003686 (PMC3744415; doi:10.1371/journal.pgen.1003686)
Supplement: Table S1 — List of the C. neoformans var neoformans strains used in this study. (DOC) [file pgen.1003686.s008.doc]

**Table S1. List of the *C. neoformans var. neoformans* strains used in this study.**

| JEC21 | *MATα* | Kwon-Chung et al., 1992 |
| --- | --- | --- |
| JEC33 | *MATα lys2* | Wickes and Edman, 1995 |
| JEC156 | *MAT***a** *ade2* *ura5* | Wickes and Edman, 1995 |
| NE116 | *MAT***a** *cas4Δ::ADE2 ura5* | This study |
| NE128 | *MAT***a** *cas3Δ::ADE2 ura5* | Moyrand et al., 2004 |
| NE291 | *MAT***a** *CAS3* *ura5* | This study |
| NE292 | *MAT***a** *cas3Δi ura5* | This study |
| NE293 | *MAT***a** *cas3Δi1-11 ura5* | This study |
| NE294 | *MAT***a** *cas3Δi2-10 ura5* | This study |
| NE295 | *MAT***a** *cas3Δi2-12 ura5* | This study |
| NE296 | *MAT***a** *cas3Δi2-6 ura5* | This study |
| NE297 | *MAT***a** *cas3Δi1-6 ura5* | This study |
| NE298 | *MAT***a** *cas3Δi1 ura5* | This study |
| NE299 | *MAT***a** *cas3Δi12 ura5* | This study |
| NE300 | *MAT***a** *cas3Δi1-10,12 ura5* | This study |
| NE449 | *MAT***a** *cas3Δi3-12 ura5* | This study |
| NE451 | *MAT***a** *cas3Δi7-10 ura5* | This study |
| NE453 | *MAT***a** *cas3Δi7-12 ura5* | This study |
| NE454 | *MAT***a** *cas3Δi7-11 ura5* | This study |
| NE456 | *MAT***a** *cas3Δi2 ura5* | This study |
| NE457 | *MAT***a** *cas3Δi1-10 ura5* | This study |
| TYCC150 | *MATα cap10Δ::ADE2 ura5* | Chang and Kwon-Chung, 1999 |
| NE750 | *MATα cap10Δ::ADE2 pNE10-URA5-cap10Δi* | This study |
| NE751 | *MATα cap10Δ::ADE2 pNE10-URA5-cap10Δi pab2Δ::NAT* | This study |
| NE175 | *MATα uxs1Δ::ADE2 ura5* | Moyrand et al, 2002 |
| NE748 | *MATα uxs1Δi ura5* | This study |
| NE749 | *MATα uxs1Δi ura5* | This study |
| NE570 | *MATα uge1Δ::NAT* | This study |
| NE758 | *MATα uge1Δ::NAT pNEO-uge1Δi* | This study |
| NE759 | *MATα uge1Δ::NAT pNEO-uge1Δi* | This study |
| NE686 | *MATα pab2Δ::NAT lys2* | This study |
| NE689 | *MAT***a** *pab2Δ::NAT cas3Δi* | This study |
| NE690 | *MAT***a** | This study |
| NE691 | *MATα* | This study |
| NE693 | *MAT***a** *cas3Δi* | This study |
| NE694 | *MAT***a** *pab2Δ::NAT* | This study |
| NE695 | *MATα pab2Δ::NAT* | This study |
| NE729 | *MATα cid14Δ::NAT lys2* | This study |
| NE731 | *MAT***a** *cid14Δ::NAT* | This study |
| NE733 | *MAT***a** | This study |
| NE735 | *MAT***a** *cid14Δ::NAT cas3Δi* | This study |
| NE736 | *MAT***a** *cas3Δi* | This study |
| NE701 | *MAT***a** *rrp6Δ::NEO ade2 ura5* | This study |
| NE704 | *MATα* | This study |
| NE705 | *MATα rrp6Δ::NEO* | This study |
| NE706 | *MAT***a** | This study |
| NE707 | *MAT***a** *rrp6Δ::NEO* | This study |
| NE715 | *MAT****a*** *pab2Δ::NAT cas3Δi ura5* | This study |
| NE730 | *MATα NAT-P_GAL7_::RRP44 lys2* | This study |
| NE743 | *MATα NAT-P_GAL7_::RRP44 cas3Δi* | This study |
| NE753 | *MATα NAT-P_GAL7_::RRP44 rrp6Δ::NEO cas3Δi* | This study |
| NE714 | *MATα pab2Δ::NAT cas3Δi ura5* | This study |
| NE739 | *MATα pab2Δ::NAT cas3Δi pNE10-URA5-GFP::PAB2* | This study |
| NE754 | *MATα pab2Δ::NAT cid14Δ::NAT CAS3* | This study |
| NE755 | *MATα pab2Δ::NAT cid14Δ::NAT cas3Δi* | This study |
| NE760 | *MAT***a** *cas4Δ::ADE2 pNE10-URA5-cas4Δi* | This study |
| NE761 | *MAT***a** *cas4Δ::ADE2 pNE10-URA5-cas4Δi* | This study |
| NE785 | *MAT***a** | This study |
| NE786 | *MAT***a** *pab2Δ::NAT* | This study |
| NE787 | *MAT***a** *NAT-P_GAL7_::RRP44* | This study |
| NE788 | *MAT***a** *pab2Δ::NAT* *NAT-P_GAL7_::RRP44* | This study |
| NE789 | *MAT***a** *cas3Δi* | This study |
| NE790 | *MAT***a** *cas3Δi pab2Δ::NAT* | This study |
| NE791 | *MAT***a** *cas3Δi NAT-P_GAL7_::RRP44* | This study |
| NE792 | *MAT***a** *cas3Δi pab2Δ::NAT* *NAT-P_GAL7_::RRP44* | This study |
| NE806 | *MAT***a** *cas3Δi12::2 ade2 ura5* | This study |
| NE807 | *MAT***a** *cas3Δi2::12 ade2 ura5* | This study |
| NE865 | *MAT***a** *cas3Δi pab2Δ::NAT NEO-P_GAL7_::XRN2 ura5* | This study |
| NE874 | *MAT***a** *cas3Δi NEO-P_GAL7_::XRN2 ade2 ura5* | This study |
