# Supplementary material for: Introns Regulate Gene Expression in Cryptococcus neoformans in a Pab2p Dependent Pathway
Source: PLoS Genet. 2013 Aug 15;9(8):e1003686. doi: 10.1371/journal.pgen.1003686 (PMC3744415; doi:10.1371/journal.pgen.1003686)
Supplement: Table S2 — List of the primers used in this study. (DOC) [file pgen.1003686.s009.doc]

**Table S2. List of the primers used in this study**

**Primers used to amplify the *PAB2* cDNA.**

PAB2cDNAf GGATCCatgcattcatcatatccatatatcacaatgtcc

PAB2cDNAr AAGCTTACCATCCTCGTCCGCGTCCCC

**Primers used for the *GFP::PAB2* construction**

Pab2ex AGATAGAACCCACCCTGTAGCC

Pab2ex2 CCTGGTCGCAATAATCCTCAG

GFP-PABf CCATCTCATTAGGCGTATTTTGAACTTCTGTATGGTGAGCAAGGGCGAGGAGC

GFP-PAB-5’3 GCTCCTCGCCCTTGCTCACCATACAGAAGTTCAAAATACGCCTAATGAGATGG

GFP-PABr CGGACATTGTGATATATGGATATGATGAATGCATCCACCACCActtgtacagctcgtccatgC

GFP-PAB-3’5 GcatggacgagctgtacaagTGGTGGTGGatgcattcatcatatccatatatcacaatgtccg

**Primers used to amplify the complete *CAS3* gene cloned in the pNE247 plasmid**

CAS3F GACAAAAAGGTGTCTGAAGCG

CAS3R GGATCCGTGAACTCATTTTGGGCGACC

**Primers used to amplify the *CAS3* cDNA.**

CAS3a AGGAGTAAGGTGAGGATATACCTCGG

CAS3AR ATGCGACGACAACCTGCAG

**Primers used to amplify the *ACT1* probe**

ACT1F CCTTGGTCATCGACAATGGC

ACT1R GATCGATACGGAGGATAGCG

**Primers used to delete PAB2**

PAB2ex AGATAGAACCCACCCTGTAGCC

PAB2-5’5 AGCATAGTCTCAACAGCCTTGG

PAB2-5’3 GTCATAGCTGTTTCCTGCCTCGTCAACGTTAGACTCATCC

MKRrPAB2 GGATGAGTCTAACGTTGACGAGGCAGGAAACAGCTATGAC

PAB2-3’5 TACAACGTCGTGACTGGGGCATGAACATGACCAACAGAGG

MKRfPAB2 CCTCTGTTGGTCATGTTCATGCCCCAGTCACGACGTTGTA

PAB2-3’3 GCTTCTGGGATCTGTAAGAGCAG

PAB2ex2 CCTGGTCGCAATAATCCTCAG

**Primers used to delete RRP6**

RRP6ex ACACTTAGGTGGAGGGTCAATG

RRP6-5’5 GAAAGATGACACTGCCCTTGTG

RRP6-5’3 GTCATAGCTGTTTCCTGCCGGAGGTAGCTTTAGACATGG

MKRrRRP6 CCATGTCTAAAGCTACCTCCGGCAGGAAACAGCTATGAC

RRP6-3’5 TACAACGTCGTGACTGGGTAACGGAGATGGCTTCACAGC

MKRfRRP6 GCTGTGAAGCCATCTCCGTTACCCAGTCACGACGTTGTA

RRP6-3’3 AGAAGTCCATGGCTAGGAGCAG

RRP5ex2 AGTATTATGGGACCGCAACAGC

**Primers used to delete *UGE1* in serotype D**

UGE1seroD-3’5 TACAACGTCGTGACTGGGAACAAGACATCCCAACGGCTAC

MKRfUGE1seroD GTAGCCGTTGGGATGTCTTGTTCCCAGTCACGACGTTGTA

UGE1seroD-3’3 ACTTACTAGCTATGCTCCCTGTGC

UGE1seroDex2 GGTAGCGTGCAGATGATGAGAG

UGE1seroD-5’5 TGAGTCATGTTCACGCTGAGG

UGE1seroD-5’3 GTCATAGCTGTTTCCTGTCCGTTGTGGCTTACCTTGAG

MKRrUGE1seroD CTCAAGGTAAGCCACAACGGACAGGAAACAGCTATGAC

**Primers used to swap the RRP44 promoter**

RRP44-5’5 CGTGGATTGGCGTATAAGAGG

RRP44-5’3 GTCATAGCTGTTTCCTGTCAAGAAGATGGGTGCCAGAG

RRP44MKRf CTCTGGCACCCATCTTCTTGACAGGAAACAGCTATGAC

GALF TACAACGTCGTGACTGGGagaagcaggtcttgtcgaac

GALR GATGCGACTTCACTGCTGCCATtctcaagaggggattgagcgctg

RRP44MKRr GATGCGACTTCACTGCTGCCATCAAGAGGGGATTGAGCGCTG

RRP44 35 CAGCGCTCAATCCCCTCTTGATGGCAGCAGTGAAGTCGCATC

RRP44-33 GTTTTGACACCTGCGAGAAGTG

RRP44f TCCGCAGAGGTTGAGACTATTG

RRP44r ACAGACAGTTGAGATCCGGACA

**Primers used to delete CID14**

CID14ex CCCGTCTCATCTCTTCTTCGTC

CID14-5’5 GATGGAGGCTGGTTCCGTATAG

CID14-5’3 GTCATAGCTGTTTCCTGGTATGGTGGAGAAGCTGGCTGT

MKRrCID14 ACAGCCAGCTTCTCCACCATACCAGGAAACAGCTATGAC

CID14-3’5 TACAACGTCGTGACTGGGGCAGAGTTCTGGGGATATGAGC

MKRfCID14 GCTCATATCCCCAGAACTCTGCCCCAGTCACGACGTTGTA

CID14-3’3 CCACCTTATGGAAATCCAGGTC

CID14ex2 TCCAAGACTGGTCTCCAACAGA

**Primers used to swap the XRN2 promoter**

pXRN2-ex TGCGGTCGGTATGGTATATGTC

pXRN2-5'5 GTAACGCCAGGGTTTTCCCAGTCACGACGGGGAGTGTGTCTTATGGAAC

pXRN2-5'3 ATCCACTTAACGTTACTGAAATCTCCTTCGCTGTCCACAGTGCATCAAGTA

GAL7-pXRN2-MKRr GGAGGATATCCGTATAATTACCATCAAGAGGGGATTGAGCGCTG

MKRf-Sc-NEO GAAGGAGATTTCAGTAACGTTAAGTGGATGGTTTATCTGTATTAACACGGAAGAG

GAL7-pXRN2-3'5 CAGCGCTCAATCCCCTCTTGATGGTAATTATACGGATATCCTCC

pXRN2-3'3 GCGGATAACAATTTCACACAGGAAACAGCCTGACGACGAATCCAGTCCATA

pXRN2-ex2 CATGGGGTCCCTTAGAACTTTG

**Primers used for qPCR**

CAS3D sens agtgaggagtctattaGatttgc

CAS3D antisens gagagagtacagggagatcg

ACT1D sens TGCGGTCGGTATGGTATATGTC

ACT1D antisens CGTTCTTGACTCTGGTGAC

Debut-CAS3_F ATGAGAATCTTGACGAGGTAG

Debut-CAS3_R gccagtgatcgcttgagatt

**Primers used for LM-PAT experiments**

CAS1-PAT gggattatgggcgttgaattgg

CAS3-PAT CTGTGTTACATCATGGCGTTGGAG

UGE1-PAT gacatcccaacggctactcctc

UXS1-PAT gagggtgccatctagatgcg

CAP10-PAT GAGGCCATGACCTACAACGG

LAC1-PAT ggcgtagcctttcgagtag

LAC2-PAT ggttgttaaacgtagaggtgc

Cas31-PAT GGGAAGGAGTTTAGGTTGATC

RPD1-PAT gccgtcgctcattctgag

RRP6-PAT GCAGGGTAATAAGTCTGGGAC

CAS4-PAT GAGCGGGTTGATCTTGTTC
